# A Liouville dynamical modelling and control framework for oesophageal cancer-induced immune response

**DOI:** 10.1098/rsos.261866

**Published:** 2026-10-28

**Authors:** Souvik Roy, Naif Abu Qarnayn, Mesfer H. F. Alajmi, Asma Alghamdi, Ibrahem Alshaoosh, Shreshtha Chaturvedi, Olusola Dehinsilu, Rodina El-Adawy, Darsh Gandhi, DeAnna Patterson, Jordan Rodriguez

**Affiliations:** ^1^ Department of Mathematics, The University of Texas at Arlington, Arlington, TX, USA; ^2^ Majmaah University, Al Majmaah, Riyadh Province, Saudi Arabia; ^3^ Najran University, Najran, Najran Province, Saudi Arabia; ^4^ University of Tabouk, Tabuk, Tabuk Province, Saudi Arabia; ^5^ UT Southwestern Medical Center, Dallas, TX, USA; ^6^ University of Maryland College Park, College Park, MD, USA

**Keywords:** Liouville optimization, sequential quadratic Hamiltonian, Pontryagin’s maximum principle, chemotherapy, immunotherapy

## Abstract

A Liouville-based framework is developed for optimizing treatment strategies in oesophageal cancer (OC) while accounting for inter-patient heterogeneity. Variability within a patient cohort is represented through uncertain initial cancer and immune cell populations, whose probability distribution is propagated by a Liouville partial differential equation while the underlying individual dynamics remains deterministic. Two optimization problems are considered. First, model parameters are estimated from population-level data using an adjoint-based projected nonlinear conjugate-gradient (NCG) method. Second, optimal time-dependent combinations of chemotherapy and immunotherapy are determined using a sequential quadratic Hamiltonian (SQH) scheme based on Pontryagin’s maximum principle (PMP). The Liouville equation is discretized using a finite-volume method designed to preserve positivity and conservation of the probability density. Numerical experiments with synthetic population data are used to demonstrate the parameter-estimation framework and the ability of the proposed optimal control strategy to determine treatment protocols that effectively modify the population-level cancer–immune response while limiting treatment exposure.

## 1. Introduction

Oesophageal cancer (OC) is the sixth leading cause of cancer mortality worldwide [1], and in the USA there were an estimated 15 530 deaths in 2021 according to Surveillance, Epidemiology, and End Results (SEER) statistics. There are two main types of OC: oesophageal squamous cell carcinoma (OSCC) and oesophageal adenocarcinoma (OAC) [2,3]. Despite current treatments, both OSCC and OAC have less than a 20% overall 5 year survival rate. Poor survival in patients with localized OC is due to rapid disease progression, metastases and chemoradiotherapy (CRT) resistance [4]. Recent clinical trials have demonstrated the overall survival benefit of adding immune checkpoint inhibitors (ICI), such as pembrolizumab and nivolumab (antibodies targeting the PD-1/PD-L1 checkpoint), to first-line combination chemotherapy in patients with unresectable or metastatic gastroesophageal cancer [5–7] leading to U.S. Food and Drugs Adminstration (FDA) approvals for ICI in this setting. Nivolumab has also been FDA–approved in the adjuvant setting for patients with residual OC or gastroesophageal junction cancer following neoadjuvant CRT and surgery [8]. Unfortunately, only a minority of OC patients respond to ICI and even in patients who initially respond, their tumours may develop adaptive resistance owing to genetic heterogeneity and diverse carcinogenic origins.

Compared to other solid tumours, OC exhibits greater heterogeneity and diversity in cancerous origins [9]. This heterogeneity includes extrinsic variations, such as gene copy number amplification and environmental influences like metabolic stress or immune response [10], and intrinsic variations driven by intracellular dynamics [11]. Such heterogeneity contributes significantly to drug resistance in OC, highlighting the need for pharmacokinetic models that account for these variations. While many clinical trials have attempted to identify predictive biomarkers to select patients who are likely to respond to ICI, the only clinically validated biomarker is PD-L1 tissue expression.

Mathematical models, particularly pharmacokinetic models, are a powerful and efficient tool for understanding OC dynamics and immune response without requiring extensive clinical trials. These models use differential equations to represent anatomical, physiological and biochemical processes influenced by cancer [12], allowing for accurate modelling and prediction of cancer progression based on patient-specific molecular characteristics. In the past, deterministic pharmacokinetic models have been used to model immune response in various cancer types, e.g. in breast cancer [13], lung cancer [14], colorectal cancer [15] and melanoma [16]. A common drawback with the previous pharmacokinetic models is that they fail to simultaneously model cancer intrinsic and extrinsic heterogeneities, thus leading to inaccurate prediction of the dynamical features as well as treatment responses. In a very recent work [17], a pharmacokinetic model for receptor tyrosine kinases (RTK) signalling pathways in OC was developed, modelling interactions between biological entities through a combination of Michaelis–Menten and mass action laws. This approach accommodates extrinsic heterogeneity, allowing different copies of the same biological entity to exhibit diverse behaviours. In this work, we consider a pharmacokinetic model that describes the population-level interaction between cancer cells and immune cells, incorporating cancer-cell proliferation, immune-mediated cancer-cell death, immune-cell activation by cancer cells, natural immune-cell inactivation and the effects of chemotherapy and immunotherapy.

A key objective of pharmacokinetic modelling in OC is to enable precise classification of patients for evaluating the effectiveness of treatments during clinical trials [18]. Randomized controlled trials are widely regarded as the standard approach for assessing how different therapies perform [19–21]. In such trials, participants are assigned to different treatment groups at random, allowing researchers to observe and compare treatment responses across varied populations. These groupings often rely on specific patient features, making it essential to capture the dynamic nature of OC progression across subgroups.

However, owing to the intrinsic heterogeneity of OC, patients within the same subgroup can exhibit substantially different baseline tumour burden, immune response and overall physiological condition. Consequently, even when subjected to the same treatment protocol, patient responses can vary considerably. Capturing this population-level variability is essential for robust parameter estimation and treatment optimization, since such biological variability is a known contributor to the failure of clinical trials [22]. In this work, we extend the deterministic pharmacokinetic model to a population-based framework that accounts for heterogeneous patient populations while preserving the underlying mechanistic dynamics.

To account for uncertainty arising from heterogeneous patient populations, we adopt a Liouville-based population framework in which each realization of the pharmacokinetic model corresponds to an individual patient, while the Liouville equation governs the evolution of the associated probability density [23]. The effects of treatment and patient responses can be framed as optimal control problems governed by Liouville equations, where the control variable corresponds to drug dosage and administration frequency. The objective is to evaluate the effectiveness of a given therapy by its ability to steer the Liouville-distributed cancer state towards a disease-free or clinically controlled state. Although the application of Liouville-based control frameworks to OC has been relatively unexplored, they offer distinct advantages [24,25]. Specifically, the Liouville formulation captures the evolution of probability densities over a range of dynamical pathways, making it particularly well suited for modelling systems with uncertain or heterogeneous initial conditions. This allows for the development of robust control strategies and adaptive feedback mechanisms that can enhance therapeutic success [26].

Liouville frameworks have been very rarely used in context of cancer modelling and simulation. The only known work is for the application of prostate cancer [27], where it was shown that this framework resulted in obtaining more robust treatment strategies even with uncertainty in the experimental data. Recent studies have demonstrated the effectiveness of feedback and optimal control strategies in tumour and disease models, including time-dependent intervention strategies based on Pontryagin’s maximum principle (PMP) [28–31]. These approaches primarily formulate control at the level of the underlying state dynamics. By contrast, the present work incorporates treatment optimization within a Liouville framework, allowing the control problem to account for the evolution of a heterogeneous population of cancer–immune states. In this work, we construct a Liouville-based dynamical system to describe the interaction between OC and the immune response. Building upon this model, we design and implement an optimal control strategy aimed at evaluating the combined efficacy of chemotherapy and immunotherapy in managing OC. To the best of our knowledge, this work represents the first application of the Liouville framework to the design of optimal treatment protocols for OC in the context of clinical trials.

For the implementation of the optimal control problem, we use a gradient-free optimal control solver, known as the sequential quadratic Hamiltonian (SQH) scheme, which is based on PMP characterization and is robust and with fast convergence. Since the PMP principle has a pointwise formulation and, thus, a local structure, the SQH algorithm implements local updates of the control function pointwise in time. The SQH scheme has been used in past in the context of a Liouville mass transport problem [23], medical imaging problems [32,33] and parabolic optimal control problems [34], and has been demonstrated to be an effective optimal control solver in the presence of non-smooth objective functionals. We use the SQH optimal control algorithm for fitting our model to the data distribution using a maximum-likelihood-based approach and obtain a robust, accurate and fast implementation of the optimal dosage problem.

The paper is organized as follows: in the next section, we describe a Liouville control framework for obtaining optimal therapies in OC. Section 3 deals with the theoretical results pertaining to the two Liouville optimization problems for parameter estimation and optimal control. We describe the SQH parameter estimation framework and SQH optimal control framework in §4. Numerical results with synthetic data are presented in §5. We end the work with conclusions (§6).

## 2. A Liouville modelling and control framework

We first consider a dynamical model for modelling the interaction between the OC cells and the activated immune cells. Denoting $y_{1}(t),y_{2}(t)$ as the number of cancer and immune cells at time t, the interactions between the cells, under the influence of the chemotherapeutic and immunotherapeutic drugs, are governed by the following system of ordinary differential equations (ODEs):

(2.1) $$\begin{array}{rl} & \frac{\text{d}{\bar{y}}_{1}}{\text{d}\bar{t}}=\underset{\text{Proliferation}}{\underbrace{{\bar{k}}_{1}{\bar{y}}_{1}\left(1-\frac{{\bar{y}}_{1}}{{\bar{L}}_{1}}\right)}}-\underset{\text{Death by immune cells}}{\underbrace{{\bar{k}}_{2}{\bar{y}}_{1}\frac{{\bar{y}}_{2}}{{\bar{y}}_{2}+{\bar{S}}_{1}}}}-\underset{\text{Death rate}}{\underbrace{{\bar{k}}_{3}{\bar{y}}_{1}}}-\underset{\text{Chemotherapy}}{\underbrace{{\bar{\nu }}_{1}{\bar{u}}_{1}(t){\bar{y}}_{1}}},\;{\bar{y}}_{1}(0)={\bar{y}}_{1}^{0},\\ & \frac{\text{d}{\bar{y}}_{2}}{\text{d}\bar{t}}=\underset{\text{Constant production}}{\underbrace{{\bar{d}}_{1}}}+\underset{\text{Activation by cancer}}{\underbrace{{\bar{d}}_{2}{\bar{y}}_{2}\frac{{\bar{y}}_{1}}{{\bar{y}}_{1}+{\bar{S}}_{2}}}}-\underset{\text{Interaction}}{\underbrace{{\bar{d}}_{3}{\bar{y}}_{1}{\bar{y}}_{2}}}-\underset{\text{Inactivation}}{\underbrace{{\bar{d}}_{4}{\bar{y}}_{2}}}+\underset{\text{Immunotherapy}}{\underbrace{{\bar{\nu }}_{2}{\bar{u}}_{2}(t)}}-\underset{\text{Chemotherapy}}{\underbrace{{\bar{\nu }}_{3}{\bar{u}}_{1}(t){\bar{y}}_{2}}},\;{\bar{y}}_{2}(0)={\bar{y}}_{2}^{0}. \end{array}$$

In equation (2.1), the cancer-cell population undergoes logistic proliferation with intrinsic growth rate ${\bar{k}}_{1}$ and carrying capacity ${\bar{L}}_{1}$. Cancer cells are eliminated through three mechanisms: immune-mediated killing, characterized by the maximum killing rate ${\bar{k}}_{2}$ and the half-saturation constant ${\bar{S}}_{1}$; natural cancer-cell death at rate ${\bar{k}}_{3}$; and chemotherapy-induced death, whose strength is determined by the treatment-response coefficient ${\bar{\nu }}_{1}$ and the chemotherapy dosage ${\bar{u}}_{1}(t)$. The activated immune-cell population has a constant production rate ${\bar{d}}_{1}$. Cancer cells stimulate additional immune-cell activation through a saturating response characterized by the activation rate ${\bar{d}}_{2}$ and half-saturation constant ${\bar{S}}_{2}$. The parameter ${\bar{d}}_{3}$ characterizes immune-cell loss arising from cancer–immune cell interactions, whereas ${\bar{d}}_{4}$ represents immune-cell inactivation owing to mechanisms such as natural death or functional exhaustion. Immunotherapy increases the activated immune-cell population through the treatment-response coefficient ${\bar{\nu }}_{2}$ and dosage ${\bar{u}}_{2}(t)$, while chemotherapy can also deplete activated immune cells, with this effect characterized by ${\bar{\nu }}_{3}$ and ${\bar{u}}_{1}(t)$. Thus, ${\bar{u}}_{1}(t)$ and ${\bar{u}}_{2}(t)$ represent the time-dependent dosages of chemotherapy and immunotherapy, respectively. A list of the biological interpretation and units of the model parameters is summarized in table 1.

**Table 1.** Parameters of the dimensional cancer–immune model, equation (2.1).

parameter	biological interpretation	units
${\bar{k}}_{1}$	intrinsic cancer-cell proliferation rate	${\mathrm{day}}^{-1}$
${\bar{L}}_{1}$	carrying capacity of the cancer-cell population	cells
${\bar{k}}_{2}$	maximum rate of immune-mediated cancer-cell killing	${\mathrm{day}}^{-1}$
${\bar{S}}_{1}$	immune-cell half-saturation constant for immune-mediated cancer-cell killing	cells
${\bar{k}}_{3}$	natural cancer-cell death rate	${\mathrm{day}}^{-1}$
${\bar{d}}_{1}$	constant production rate of activated immune cells	cells $\times \;{\mathrm{day}}^{-1}$
${\bar{d}}_{2}$	maximum rate of cancer-induced activation of immune cells	${\mathrm{day}}^{-1}$
${\bar{S}}_{2}$	cancer-cell half-saturation constant for immune-cell activation	cells
${\bar{d}}_{3}$	cancer–immune interaction coefficient governing immune-cell depletion	$(\mathrm{cell}\times \mathrm{day}{)}^{-1}$
${\bar{d}}_{4}$	natural inactivation/death rate of activated immune cells	${\mathrm{day}}^{-1}$
${\bar{\nu }}_{1}$	chemotherapy efficacy coefficient for cancer-cell killing	$\mathrm{kg}\;{\mathrm{mg}}^{-1}{\mathrm{day}}^{-1}$
${\bar{\nu }}_{2}$	immunotherapy efficacy coefficient for production/activation of immune cells	cells $\times \;\mathrm{kg}\;{\mathrm{mg}}^{-1}{\mathrm{day}}^{-1}$
${\bar{\nu }}_{3}$	chemotherapy efficacy coefficient for immune-cell depletion	$\mathrm{kg}\;{\mathrm{mg}}^{-1}{\mathrm{day}}^{-1}$
${\bar{u}}_{1}(t)$	time-dependent chemotherapy dosage	mgkg^–1^
${\bar{u}}_{2}(t)$	time-dependent immunotherapy dosage	mgkg^–1^

For stabilization and scalability of the numerical algorithms, we non-dimensionalize the above ODE system using the following non-dimensionalized state and time variables, and parameters:

(2.2) $$\begin{array}{rl} & y_{1}=M_{1}{\bar{y}}_{1},\;y_{2}=M_{2}{\bar{y}}_{2},\;t=M_{3}\bar{t},\\ & k_{1}=\frac{M_{1}}{M_{3}}{\bar{k}}_{1},\;k_{2}=\frac{1}{M_{3}}{\bar{k}}_{2},\;k_{3}=\frac{1}{M_{3}}{\bar{k}}_{3}\\ \text{and } & d_{1}=\frac{M_{2}}{M_{3}}{\bar{d}}_{1},\;d_{2}=\frac{1}{M_{3}}{\bar{d}}_{2},\;d_{3}=\frac{1}{M_{1}M_{3}}{\bar{d}}_{3},\;d_{4}=\frac{1}{M_{3}}{\bar{d}}_{4}. \end{array}$$

The resulting non-dimensional ODE system is expressed as follows:

(2.3) $$\begin{array}{rl} & \frac{\text{d}y_{1}}{\text{d}t}=k_{1}y_{1}\left(1-\frac{y_{1}}{L_{1}}\right)-k_{2}y_{1}\frac{y_{2}}{y_{2}+S_{1}}-k_{3}y_{1}-\nu_{1}u_{1}(t)y_{1},\;y_{1}(0)=y_{1}^{0}\\ \text{and } & \frac{\text{d}y_{2}}{\text{d}t}=d_{1}+d_{2}y_{2}\frac{y_{1}}{y_{1}+S_{2}}-d_{3}y_{1}y_{2}-d_{4}y_{2}+\nu_{2}u_{2}(t)-\nu_{3}u_{1}(t)y_{2},\;y_{2}(0)=y_{2}^{0}. \end{array}$$

Using a compact notation, we can write the ODE system equation (2.3) as follows:

(2.4) $$\begin{array}{rl} & \text{d}X=b(X,\theta ,u)\;\text{d}t,\;X(0)=X_{0}, \end{array}$$

where $X(t)=(y_{1}(t),y_{2}(t){)}^{T}$ and $\theta =(k_{2},k_{3},d_{2},d_{3})\in T_{\text{ad}}=\{\theta \in R^{4}:0\leq \theta_{i}\leq T_{i}\}$ is the set of patient-specific parameters that need to be estimated for fitting the model.

### 2.1. Qualitative properties of the pharmacokinetic model

We first establish several qualitative properties of the pharmacokinetic model, equation (2.4).

**Theorem 2.1.**

*Suppose the control functions satisfy*

$$u_{i}\in L^{\infty }(0,T)\;and\;0\leq u_{i}(t)\leq D_{i},\;i=1,2,$$

*and assume that the initial conditions satisfy $y_{1}^{0}\geq 0,\;y_{2}^{0}\geq 0.$ Then, the ODE system equation (2.3) admits a unique solution $\left(y_{1},y_{2})\in C^{1}([0,T];R^{2}\right)$.*

Proof.
Since every term appearing in equation (2.3) is continuously differentiable with respect to $\left(y_{1},y_{2}\right)$, the vector field is locally Lipschitz continuous. Therefore, the classical Picard–Lindelöf theorem guarantees existence and uniqueness of a local solution. The boundedness result established below excludes finite-time blow-up and therefore the solution extends uniquely to the entire interval [0,T].

**Proposition 1** (Positivity). *Let $\left(y_{1},y_{2}\right)$ denote the solution of equation (2.3). If $y_{1}^{0}\geq 0,\;y_{2}^{0}\geq 0$, then $y_{1}(t)\geq 0,\;y_{2}(t)\geq 0$, for every t≥0.*

Proof.
Suppose first that $y_{1}(t)=0$. Then from equation (2.3), we have

$$\frac{\text{d}y_{1}}{\text{d}t}=0,$$

showing that the vector field is tangent to the boundary $y_{1}=0$. Hence, the solution cannot cross into the region $y_{1}<0$. Similarly, when $y_{2}(t)=0$,

$$\frac{\text{d}y_{2}}{\text{d}t}=d_{1}+\nu_{2}u_{2}(t)\geq 0,$$

since $d_{1}>0$ and $u_{2}(t)\geq 0$. Therefore, the vector field points into the positive quadrant and the solution cannot become negative. Hence, the non-negative quadrant

$$R_{+}^{2}=\{(y_{1},y_{2})\in R^{2}:\;y_{1}\geq 0,\;y_{2}\geq 0\}$$

is positively invariant under the dynamics of equation (2.3). Consequently, any solution that starts with non-negative initial conditions remains non-negative.

**Proposition 2** (Boundedness). *Every solution of equation (2.3) remains bounded on [0,T].*

Proof.
Since

$$-k_{2}y_{1}\frac{y_{2}}{y_{2}+S_{1}}\leq 0,\;-k_{3}y_{1}\leq 0\;\text{and}\;-\nu_{1}u_{1}y_{1}\leq 0,$$

we obtain

$$\frac{\text{d}y_{1}}{\text{d}t}\leq k_{1}y_{1}\left(1-\frac{y_{1}}{L_{1}}\right).$$

The comparison principle therefore implies

$$0\leq y_{1}(t)\leq \max\{y_{1}^{0},L_{1}\},$$

for every t≥0. Since $y_{1}$ is bounded, there exists a constant $C_{1}>0$ such that

$$\frac{y_{1}}{y_{1}+S_{2}}\leq 1\;\text{and}\;d_{1}+d_{2}y_{2}\frac{y_{1}}{y_{1}+S_{2}}\leq d_{1}+d_{2}y_{2}.$$

Hence,

$$\frac{\text{d}y_{2}}{\text{d}t}\leq d_{1}+\nu_{2}D_{2}+(d_{2}-d_{4})y_{2}.$$

By Gronwall’s inequality,

$$y_{2}(t)\leq C,$$

where C>0 depends only on the model parameters, the controls and the initial data. Consequently, both state variables remain bounded throughout the simulation interval.

As a consequence of the previous propositions, every biologically admissible trajectory remains inside a positively invariant bounded subset of the first quadrant. Therefore, there exists a constant A>0 such that

$$0\leq y_{1}(t)\leq A\text{and}\;0\leq y_{2}(t)\leq A,$$

for all t∈[0,T].

### 2.2. Equilibria and local stability of the untreated model

We next study the equilibria and their local stability for the untreated pharmacokinetic model. Setting $u_{1}=u_{2}=0$ in equation (2.3) gives

(2.5) $$\begin{array}{rl} \frac{\text{d}y_{1}}{\text{d}t} & =k_{1}y_{1}\left(1-\frac{y_{1}}{L_{1}}\right)-k_{2}y_{1}\frac{y_{2}}{y_{2}+S_{1}}-k_{3}y_{1}\\ \text{and }\frac{\text{d}y_{2}}{\text{d}t} & =d_{1}+d_{2}y_{2}\frac{y_{1}}{y_{1}+S_{2}}-d_{3}y_{1}y_{2}-d_{4}y_{2}. \end{array}$$

For convenience, we write equation (2.5) as

$$\frac{\text{d}y_{1}}{\text{d}t}=F_{1}(y_{1},y_{2})\;\text{and}\;\frac{\text{d}y_{2}}{\text{d}t}=F_{2}(y_{1},y_{2}).$$

An equilibrium $E^{*}=(y_{1}^{*},y_{2}^{*})$ satisfies

$$F_{1}(y_{1}^{*},y_{2}^{*})=0\;\text{and}\;F_{2}(y_{1}^{*},y_{2}^{*})=0.$$

#### 2.2.1. Tumour-free equilibrium

Setting $y_{1}^{*}=0$ in the second equilibrium equation gives

$$d_{1}-d_{4}y_{2}^{*}=0.$$

Therefore, the tumour-free equilibrium is

(2.6) $$E_{0}=\left(0,\frac{d_{1}}{d_{4}}\right).$$

Biologically, this equilibrium represents the absence of cancer cells together with the baseline activated immune-cell population maintained by the balance between constant immune-cell production and natural inactivation.

#### 2.2.2. Tumour-present equilibria

For a tumour-present equilibrium, $y_{1}^{*}>0$. Dividing the first equilibrium equation by $y_{1}^{*}$ gives

(2.7) $$k_{1}\left(1-\frac{y_{1}^{*}}{L_{1}}\right)-k_{2}\frac{y_{2}^{*}}{y_{2}^{*}+S_{1}}-k_{3}=0.$$

The second equilibrium equation can be written as

(2.8) $$d_{1}+y_{2}^{*}\left(d_{2}\frac{y_{1}^{*}}{y_{1}^{*}+S_{2}}-d_{3}y_{1}^{*}-d_{4}\right)=0.$$

If

$$d_{4}+d_{3}y_{1}^{*}-d_{2}\frac{y_{1}^{*}}{y_{1}^{*}+S_{2}}>0,$$

equation (2.8) yields

(2.9) $$y_{2}^{*}=\frac{d_{1}}{d_{4}+d_{3}y_{1}^{*}-d_{2}\frac{y_{1}^{*}}{y_{1}^{*}+S_{2}}}.$$

Substituting equation (2.9) into equation (2.7) gives a scalar nonlinear equation for $y_{1}^{*}$. Because of the nonlinear saturation terms, an explicit closed-form expression is generally unavailable. The biologically admissible tumour-present equilibria are therefore the positive solutions of equations (2.7)–(2.8).

#### 2.2.3. Local stability

The Jacobian matrix of the untreated system equation (2.5) is

(2.10) $$J(y_{1},y_{2})=\left(\begin{array}{cc} J_{11}(y_{1},y_{2}) & J_{12}(y_{1},y_{2})\\ J_{21}(y_{1},y_{2}) & J_{22}(y_{1},y_{2}) \end{array}\right),$$

where

$$\begin{array}{rl} J_{11}(y_{1},y_{2}) & =k_{1}\left(1-\frac{2y_{1}}{L_{1}}\right)-k_{2}\frac{y_{2}}{y_{2}+S_{1}}-k_{3},\\ J_{12}(y_{1},y_{2}) & =-\frac{k_{2}S_{1}y_{1}}{(y_{2}+S_{1}{)}^{2}},\\ J_{21}(y_{1},y_{2}) & =\frac{d_{2}S_{2}y_{2}}{(y_{1}+S_{2}{)}^{2}}-d_{3}y_{2}\\ \text{and }J_{22}(y_{1},y_{2}) & =d_{2}\frac{y_{1}}{y_{1}+S_{2}}-d_{3}y_{1}-d_{4}. \end{array}$$

**Proposition 3.**

*The tumour-free equilibrium*

$$E_{0}=\left(0,\frac{d_{1}}{d_{4}}\right)$$

*is locally asymptotically stable if*

(2.11) $$k_{1}-k_{3}-k_{2}\frac{d_{1}}{d_{1}+d_{4}S_{1}}<0,$$

*and it is unstable if the left-hand side of equation (2.11) is positive.*

Proof.
Evaluating the Jacobian matrix equation (2.10) at $E_{0}$ gives

$$J(E_{0})=\left(\begin{array}{cc} k_{1}-k_{3}-k_{2}\frac{d_{1}}{d_{1}+d_{4}S_{1}} & 0\\ \frac{d_{1}}{d_{4}}\left(\frac{d_{2}}{S_{2}}-d_{3}\right) & -d_{4} \end{array}\right).$$

Since $J(E_{0})$ is lower triangular, its eigenvalues are

$$\lambda_{1}=k_{1}-k_{3}-k_{2}\frac{d_{1}}{d_{1}+d_{4}S_{1}}\;\text{and}\;\lambda_{2}=-d_{4}.$$

Because $d_{4}>0$, one has $\lambda_{2}<0$. Therefore, both eigenvalues are negative precisely when equation (2.11) holds.

Condition (2.11) can equivalently be written as

$$k_{1}<k_{3}+k_{2}\frac{d_{1}}{d_{1}+d_{4}S_{1}}.$$

Thus, the tumour-free state is locally asymptotically stable when the effective cancer-cell proliferation rate is smaller than the combined natural and immune-mediated cancer-cell removal rates near zero tumour burden.

**Proposition 4.**

*Let $E_{+}=(y_{1}^{*},y_{2}^{*})$ be a biologically admissible tumour-present equilibrium satisfying equations (2.7)–(2.8). Then, the Jacobian matrix evaluated at $E_{+}$ is*

$$J(E_{+})=\left(\begin{array}{cc} -\frac{k_{1}}{L_{1}}y_{1}^{*} & -\frac{k_{2}S_{1}y_{1}^{*}}{(y_{2}^{*}+S_{1}{)}^{2}}\\ \frac{d_{2}S_{2}y_{2}^{*}}{(y_{1}^{*}+S_{2}{)}^{2}}-d_{3}y_{2}^{*} & d_{2}\frac{y_{1}^{*}}{y_{1}^{*}+S_{2}}-d_{3}y_{1}^{*}-d_{4} \end{array}\right).$$

*Consequently,*

(2.12) $$\mathrm{tr}(J(E_{+}))=y_{1}^{*}\left(-\frac{k_{1}}{L_{1}}+\frac{d_{2}}{y_{1}^{*}+S_{2}}-d_{3}\right)-d_{4},$$

*and*

(2.13) $$\begin{array}{rl} \det(J(E_{+}))= & -\frac{k_{1}}{L_{1}}y_{1}^{*}\left(d_{2}\frac{y_{1}^{*}}{y_{1}^{*}+S_{2}}-d_{3}y_{1}^{*}-d_{4}\right)\\ & +\frac{k_{2}S_{1}y_{1}^{*}}{(y_{2}^{*}+S_{1}{)}^{2}}\left(\frac{d_{2}S_{2}y_{2}^{*}}{(y_{1}^{*}+S_{2}{)}^{2}}-d_{3}y_{2}^{*}\right). \end{array}$$

*Therefore, the tumour-present equilibrium $E_{+}$ is locally asymptotically stable if*

(2.14) $$\mathrm{tr}(J(E_{+}))<0\;and\;\det(J(E_{+}))>0.$$

*If*

$$\det(J(E_{+}))<0,$$

*then $E_{+}$ is a saddle point and is therefore unstable.*

Proof.
Evaluating the Jacobian matrix equation (2.10) at the tumour-present equilibrium gives

$$J_{11}(E_{+})=k_{1}\left(1-\frac{2y_{1}^{*}}{L_{1}}\right)-k_{2}\frac{y_{2}^{*}}{y_{2}^{*}+S_{1}}-k_{3}.$$

Since $y_{1}^{*}>0$, the first equilibrium equation implies

$$k_{1}\left(1-\frac{y_{1}^{*}}{L_{1}}\right)=k_{2}\frac{y_{2}^{*}}{y_{2}^{*}+S_{1}}+k_{3}.$$

Hence,

$$J_{11}(E_{+})=-\frac{k_{1}}{L_{1}}y_{1}^{*}.$$

Substitution of this identity into equation (2.10) gives the stated form of $J(E_{+})$. Its trace is

$$\begin{array}{rl} \mathrm{tr}(J(E_{+})) & =-\frac{k_{1}}{L_{1}}y_{1}^{*}+d_{2}\frac{y_{1}^{*}}{y_{1}^{*}+S_{2}}-d_{3}y_{1}^{*}-d_{4}\\ & =y_{1}^{*}\left(-\frac{k_{1}}{L_{1}}+\frac{d_{2}}{y_{1}^{*}+S_{2}}-d_{3}\right)-d_{4}, \end{array}$$

while direct evaluation of

$$\det(J(E_{+}))=J_{11}(E_{+})J_{22}(E_{+})-J_{12}(E_{+})J_{21}(E_{+})$$

gives equation (2.13). The characteristic polynomial associated with $J(E_{+})$ is

$$\lambda^{2}-\mathrm{tr}(J(E_{+}))\lambda +\det(J(E_{+}))=0.$$

Therefore, by the Routh–Hurwitz criterion for a two-dimensional system, both eigenvalues have negative real parts if and only if

$$\mathrm{tr}(J(E_{+}))<0\;\text{and}\;\det(J(E_{+}))>0.$$

Thus, $E_{+}$ is locally asymptotically stable under these conditions. If $\det(J(E_{+}))<0$, the eigenvalues have opposite signs, so $E_{+}$ is a saddle point and is unstable.

The trace condition equation (2.12) shows that local stability depends on the balance among logistic saturation, cancer-induced immune activation, cancer–immune interaction and natural immune-cell inactivation. In particular, stronger logistic saturation, immune-cell loss through interaction with cancer cells and natural immune inactivation contribute negatively to the trace, whereas cancer-induced immune activation contributes positively.

### 2.3. Ensemble dynamics and the Liouville equation

In clinical studies of OC, patients receiving the same treatment regimen often exhibit substantial variability in baseline tumour burden, immune response and overall physiological condition. Although individuals within the same treatment group are assumed to follow the same underlying pharmacokinetic dynamics, differences in their initial physiological states lead to heterogeneous treatment responses. Consequently, the previously described deterministic framework is inadequate for representing this inter-patient variability. Instead, it is natural to treat $y_{1}^{0}$ and $y_{2}^{0}$ as random variables representing different individuals within the population, sampled from an appropriate probability distribution. This inherently renders the state variables $y_{1}$ and $y_{2}$ random variables. Accordingly, the ODE system equation (2.3) describes the deterministic trajectory of an individual patient within the ensemble dynamics of OC, with each realization corresponding to a distinct set of initial conditions.

Although Itô stochastic differential equation models and their associated Fokker–Planck equations provide a natural framework for modelling intrinsic biological fluctuations [35], they require the specification and estimation of additional diffusion coefficients that characterize stochastic forcing. In clinical studies of OC, longitudinal measurements of tumour burden and immune response are typically sparse and available only at a limited number of clinical visits, making reliable estimation of these stochastic diffusion parameters difficult (e.g. [36]). Since the objective of the present work is to quantify uncertainty arising from heterogeneous baseline patient characteristics rather than intrinsic stochastic fluctuations during disease progression, we assume that each patient evolves according to the same deterministic pharmacokinetic model while the uncertainty is represented through a probability distribution of the initial conditions. Under these assumptions, the evolution of the corresponding probability density is naturally governed by the Liouville equation.

Let $p(x_{1},x_{2},t)$ denote the joint probability density function (PDF) associated with $y_{1},y_{2}$, such that

$$P(y_{1}(t)=x_{1},y_{2}(t)=x_{2})=p(x_{1},x_{2},t).$$

The ensemble dynamics of equation (2.3) are then governed by the following Liouville equation: let $x=(x_{1},x_{2})$ with

x∈Ω=(0,A)×(0,A).

Then, we have

(2.15) $$\begin{array}{rl} & \frac{\text{d}p}{\text{d}t}+\nabla \cdot [b(x,\theta ,u(t))p(x,t)]=0,\;(x,t)\in \Omega \times (0,T],\\ & p(x,0)=p_{0}(x),\;x\in \Omega , \end{array}$$

subject to the homogeneous Dirichlet boundary condition

(2.16) p(x,t)=0,x∈∂Ωandt∈(0,T].

The controls $u=(u_{1},u_{2})$ belong to the following admissible set:

$$U_{\text{ad}}=\{u\in (L^{2}([0,T]){)}^{2}:0\leq u_{i}(t)\leq D_{i},\;D_{i}>0,\;\forall t\in [0,T],\;i=1,2\}\text{,}$$

with $D_{i}$ representing the maximum tolerable dosages, $b(x,u)=\left(b_{1}(x,u),b_{2}(x,u)\right)$ with

$$\begin{array}{rl} & b_{1}(x,u)=k_{1}x_{1}\left(1-\frac{x_{1}}{L_{1}}\right)-k_{2}x_{1}\frac{x_{2}}{x_{2}+S_{1}}-k_{3}x_{1}-\nu_{1}u_{1}(t)x_{1}\\ \text{and } & b_{2}(x,u)=d_{1}+d_{2}x_{2}\frac{x_{1}}{x_{1}+S_{2}}-d_{3}x_{1}x_{2}-d_{4}x_{2}+\nu_{2}u_{2}(t)-\nu_{3}u_{1}(t)x_{2}. \end{array}$$

The initial condition at t=0 is given by $p(x,0)=p_{0}(x)$, and

$$\nabla \equiv \left(\frac{\partial }{\partial x_{1}},\frac{\partial }{\partial x_{2}}\right).$$

By the positivity and boundedness results established above, every biologically admissible solution of equation (2.3) remains confined to a positively invariant bounded region. Therefore, the probability density associated with $\left(y_{1},y_{2}\right)$ evolves in the bounded domain

Ω=(0,A)×(0,A),

which justifies the choice of a bounded spatial domain for the Liouville equation. By the positivity and boundedness results established above, every biologically admissible solution of equation (2.3) remains confined to a positively invariant bounded region. We therefore choose A>0 sufficiently large so that this invariant region, together with the support of the initial probability density, is contained strictly inside

Ω=(0,A)×(0,A).

Since the Liouville equation transports the initial probability density along the characteristics of equation (2.3), its support remains inside this invariant region for all t∈[0,T]. Consequently, the probability density vanishes at the outer computational boundary ∂Ω, which is consistent with the homogeneous Dirichlet boundary condition equation (2.16).

### 2.4. Optimal therapies using Liouville algorithm

We now present the algorithm used to compute the optimal combination of chemotherapeutic and immunotherapeutic drug dosages.

— We first estimate the patient specific unknown parameter vector θ, given the values of p(x,t) at specific time instants $t_{1},\cdots ,t_{N}$ as $p_{i}^{*}(x),\;i=1,\cdots ,N$. For this purpose, we solve the following optimization problem:
  (2.17) $$\begin{array}{rl} \theta^{*}=\underset{\theta \in T_{\text{ad}}}{\arg\min}J_{1}(p,\theta ):= & \;\alpha \int_{Q}p^{*}(x,t)\log\left(\frac{p^{*}(x,t)}{p(x,t)}\right)\;\text{d}x\text{d}t+\frac{\beta }{2}\|\theta {\|}_{l^{2}}^{2}, \end{array}$$
  subject to the Liouville system equation (2.15) with $u_{1}=u_{2}=0$, where $p^{*}(x,t)$ is the data function formed by interpolating the patient data distributions $p_{i}^{*}(x)$ and Q=Ω×(0,T). The distributions $p_{i}^{*}$ are chosen to reflect the uncertainty in the measurement of the data x at time $t_{i}$. We remark that when β=0, the objective functional corresponds to minimizing a regularized Kullback–Leibler (KL) divergence (relative entropy) between the observed and modelled densities, equivalent to a maximum likelihood estimation of θ.
— We then formulate a Liouville optimal control problem where the goal is to use the chemotherapy and the immunotherapy to drive the PDF p to a desired controlled state $p^{d}$; it is mathematically formulated as follows:
  (2.18) $$\begin{array}{rl} \min_{u\in U_{\text{ad}}}J_{2}(p,u) & :=\frac{\nu }{2}\int_{Q}(p(x,t)-p^{d}(x,t){)}^{2}\;\text{d}x\text{d}t+\frac{\gamma_{1}}{2}\int_{0}^{T}u_{1}^{2}(t)\;\text{d}t+\beta_{1}\int_{0}^{T}|u_{1}(t)|\;\text{d}t\\ & +\frac{\gamma_{2}}{2}\int_{0}^{T}u_{2}^{2}(t)\;\text{d}t+\beta_{2}\int_{0}^{T}|u_{2}(t)|\;\text{d}t, \end{array}$$
  subject to the Liouville system equation (2.15), where $p^{d}$ is a target PDF at the final time T. The first term in equation (2.18) is the least-squares tracking term to ensure the controlled distribution p is close to the target distribution $p^{d}$. The other terms represent the $L^{2}-L^{1}$ costs of the control. While the $L^{2}$ cost ensures there are no rapid spikes in the control values, the $L^{1}$ cost introduces sparsity patterns in the control, representing no treatment stage. At the end of the second step, we obtain the optimal drug concentration and the dosage profile over time.

## 3. Theory of the Liouville parameter estimation and control problems

In this section, we present theoretical results concerning the Liouville system equation (2.15) and the existence of solutions to the optimization problems equations (2.17)–(2.18). For this purpose, we denote the Liouville system equation (2.15) compactly as $E(p_{0},\theta ,u)=0$. We begin by addressing the existence of weak solutions to equation (2.15), as stated in the following proposition. The proof follows similar arguments to those developed for transport equations on bounded domains in [37,38]. For corresponding results on unbounded domains, we refer to [39].

**Proposition 5.**

*Let $p_{0}\in H^{1}(\Omega )$ with $p_{0}\geq 0$, and let $\theta \in T_{\text{ad}}$. Then, there exists a unique non-negative solution to $E(p_{0},\theta ,u)=0$, given by $p\in C([0,T];H^{1}(\Omega ))$.*

We now prove the following proposition, which establishes the conservation of total probability for the Liouville system.

**Proposition 6.**

*The Liouville system described in equation (2.15) preserves mass; that is, it is conservative.*

Proof.
Let $\psi \in H^{1}(\Omega )$ be a test function. Multiplying both sides of equation (2.15) by ψ, integrating over Ω and applying integration by parts along with the Dirichlet boundary condition, we obtain

(3.1) $$\int_{\Omega }\frac{\partial p}{\partial t}\;\psi \;\text{d}x=\int_{\Omega }(bp)\cdot \nabla \psi \;\text{d}x.$$

Choosing ψ=1 in equation (3.1), we observe that the right-hand side vanishes, yielding

$$\frac{\text{d}}{\text{d}t}\int_{\Omega }p(x,t)\;\text{d}x=0.$$

This implies that the total mass is conserved over time, i.e.

$$\int_{\Omega }p(x,t)\;\text{d}x=\int_{\Omega }p_{0}(x)\;\text{d}x=1,\;\forall t\in (0,T],$$

which completes the proof.

We next establish a stability property of the Liouville system.

**Proposition 7.**

*Let p be the solution to the Liouville system equation (2.15). Then, the following stability estimate holds:*

(3.2) $$\|p(t){\|}_{L^{2}(\Omega )}\leq \|p_{0}{\|}_{H^{1}(\Omega )}\exp\left(\int_{0}^{T}\frac{\|\nabla \cdot b{\|}_{L^{\infty }(\Omega )}}{2}\;\text{d}t\right),\;\forall t\in [0,T].$$

Proof.
We can write equation (3.1) as

(3.3) $$\begin{array}{rl} \int_{\Omega }\frac{\partial p}{\partial t}\psi \text{d}x & =-\int_{\Omega }\nabla \cdot (bp)\psi \;\text{d}x. \end{array}$$

Choosing ψ=p(⋅,t) in equation (3.3), we obtain

(3.4) $$\frac{\partial }{\partial t}\|p(t){\|}_{L^{2}(\Omega )}^{2}=-2\int_{\Omega }\nabla \cdot (bp)p\;\text{d}x=-2\int_{\Omega }(\nabla \cdot b)p^{2}+bp\cdot \nabla p\;\text{d}x.$$

Now,

$$\int_{\Omega }bp\cdot \nabla p\;\text{d}x=-\int_{\Omega }\nabla \cdot (bp)p\;\text{d}x=-\int_{\Omega }(\nabla \cdot b)p^{2}+bp\cdot \nabla p\;\text{d}x.$$

This implies

$$\int_{\Omega }bp\cdot \nabla p\;\text{d}x=-\frac{1}{2}\int_{\Omega }(\nabla \cdot b)p^{2}\;\text{d}x.$$

Thus,

$$\frac{\partial }{\partial t}\|p(t){\|}_{L^{2}(\Omega )}^{2}=-\int_{\Omega }(\nabla \cdot b)p^{2}\;\text{d}x.$$

Using Gronwall’s inequality, we arrive at the desired result.

We next establish continuous dependence of the Liouville solution on the model parameters and control variables. Let

$$X=R^{4}\times (L^{2}(0,T){)}^{2}$$

be equipped with the norm

$$\|(\theta ,u){\|}_{X}=|\theta |+\|u{\|}_{(L^{2}(0,T){)}^{2}}.$$

**Proposition 8.**

*Let*

$$\Lambda :T_{\text{ad}}\times U_{\text{ad}}⟶C([0,T];L^{2}(\Omega ))\;and\;(\theta ,u)⟼p=\Lambda (\theta ,u)$$

*denote the parameter-to-state mapping associated with the Liouville equation (2.15). Assume that the corresponding velocity fields satisfy*

$$b(\theta ,u)\in L^{2}(0,T;W^{1,\infty }(\Omega ;R^{2}))$$

*uniformly for $(\theta ,u)\in T_{\text{ad}}\times U_{\text{ad}}$ and that there exists a constant $C_{b}>0$ such that*

(3.5) $$\|b(\theta_{1},u_{1})-b(\theta_{2},u_{2}){\|}_{L^{2}(0,T;W^{1,\infty }(\Omega ))}\leq C_{b}\left(|\theta_{1}-\theta_{2}|+\|u_{1}-u_{2}{\|}_{(L^{2}(0,T){)}^{2}}\right).$$

*Assume further that the corresponding solutions are uniformly bounded in $C([0,T];H^{1}(\Omega ))$. Then, Λ is continuous. In particular, if*

$$p_{i}=\Lambda (\theta_{i},u_{i}),\;i=1,2$$

*are generated from the same initial density $p_{0}$, then*

(3.6) $$\|p_{1}-p_{2}{\|}_{C([0,T];L^{2}(\Omega ))}\leq C\left(|\theta_{1}-\theta_{2}|+\|u_{1}-u_{2}{\|}_{(L^{2}(0,T){)}^{2}}\right),$$

*where C>0 is independent of the particular admissible pairs $\left(\theta_{i},u_{i}\right)$.*

Proof.
Let

$$b_{i}=b(x,t,\theta_{i},u_{i}),\;p_{i}=\Lambda (\theta_{i},u_{i}),\;i=1,2,$$

and set

$$w=p_{1}-p_{2}.$$

The two states satisfy

$$\partial_{t}p_{i}+\nabla \cdot (b_{i}p_{i})=0.$$

Subtracting the aforementioned equations, one in $p_{1}$ and the other in $p_{2}$, gives

(3.7) $$\partial_{t}w+\nabla \cdot (b_{1}w)=-\nabla \cdot \left((b_{1}-b_{2})p_{2}\right),$$

with

w(x,0)=0.

Since both solutions satisfy the same homogeneous Dirichlet boundary condition, one also has

w=0on∂Ω×(0,T].

Multiplying equation (3.7) by w and integrating over Ω gives

$$\frac{1}{2}\frac{\text{d}}{\text{d}t}\|w(t){\|}_{L^{2}(\Omega )}^{2}=-\int_{\Omega }\nabla \cdot (b_{1}w)\;w\;\text{d}x-\int_{\Omega }\nabla \cdot \left((b_{1}-b_{2})p_{2}\right)w\;\text{d}x.$$

Using the homogeneous boundary condition and the same energy calculation as in proposition 7,

$$-\int_{\Omega }\nabla \cdot (b_{1}w)\;w\;\text{d}x=-\frac{1}{2}\int_{\Omega }(\nabla \cdot b_{1})w^{2}\;\text{d}x,$$

and therefore

$$\left|\int_{\Omega }\nabla \cdot (b_{1}w)\;w\;\text{d}x\right|\leq \frac{1}{2}\|\nabla \cdot b_{1}{\|}_{L^{\infty }(\Omega )}\|w{\|}_{L^{2}(\Omega )}^{2}.$$

For the second term, define

$$\delta b=b_{1}-b_{2}.$$

Since

$$\nabla \cdot (\delta b\;p_{2})=(\nabla \cdot \delta b)p_{2}+\delta b\cdot \nabla p_{2},$$

we obtain

$$\begin{array}{rl} \|\nabla \cdot (\delta b\;p_{2}){\|}_{L^{2}(\Omega )} & \leq \|\nabla \cdot \delta b{\|}_{L^{\infty }(\Omega )}\|p_{2}{\|}_{L^{2}(\Omega )}\\ & \;+\|\delta b{\|}_{L^{\infty }(\Omega )}\|\nabla p_{2}{\|}_{L^{2}(\Omega )}\\ & \leq C\|\delta b{\|}_{W^{1,\infty }(\Omega )}\|p_{2}{\|}_{H^{1}(\Omega )}. \end{array}$$

Hence, by the Cauchy–Schwarz and Young inequalities,

$$\begin{array}{rl} \left|\int_{\Omega }\nabla \cdot (\delta b\;p_{2})w\;\text{d}x\right| & \leq C\|\delta b{\|}_{W^{1,\infty }(\Omega )}\|p_{2}{\|}_{H^{1}(\Omega )}\|w{\|}_{L^{2}(\Omega )}\\ & \leq \frac{1}{2}\|w{\|}_{L^{2}(\Omega )}^{2}+C\|\delta b{\|}_{W^{1,\infty }(\Omega )}^{2}\|p_{2}{\|}_{H^{1}(\Omega )}^{2}. \end{array}$$

Combining the preceding estimates yields

$$\frac{\text{d}}{\text{d}t}\|w(t){\|}_{L^{2}(\Omega )}^{2}\leq C\left(1+\|\nabla \cdot b_{1}(t){\|}_{L^{\infty }(\Omega )}\right)\|w(t){\|}_{L^{2}(\Omega )}^{2}+C\|\delta b(t){\|}_{W^{1,\infty }(\Omega )}^{2}\|p_{2}(t){\|}_{H^{1}(\Omega )}^{2}.$$

Since $p_{2}$ is uniformly bounded in $C([0,T];H^{1}(\Omega ))$ and $b_{1}\in L^{2}(0,T;W^{1,\infty }(\Omega ))$, Gronwall’s inequality gives

$$\|w{\|}_{C([0,T];L^{2}(\Omega ))}^{2}\leq C\|\delta b{\|}_{L^{2}(0,T;W^{1,\infty }(\Omega ))}^{2}.$$

Taking square roots and using equation (3.5) gives

$$\|p_{1}-p_{2}{\|}_{C([0,T];L^{2}(\Omega ))}\leq C\left(|\theta_{1}-\theta_{2}|+\|u_{1}-u_{2}{\|}_{(L^{2}(0,T){)}^{2}}\right).$$

This proves the continuity of Λ.

We next establish the Fréchet differentiability of the parameter-to-state mapping. Since the admissible set $T_{\text{ad}}\times U_{\text{ad}}$ is not open, differentiability is understood for an extension of Λ to an open neighbourhood of $T_{\text{ad}}\times U_{\text{ad}}$ in

$$X=R^{4}\times (L^{2}(0,T){)}^{2},$$

and the resulting derivative is then restricted to admissible perturbations.

**Proposition 9.**

*Let*

$$\Lambda :X⊃O⟶C([0,T];L^{2}(\Omega )),$$

*where O is an open neighbourhood of $T_{\text{ad}}\times U_{\text{ad}}$. Assume that*

(θ,u)⟼b(θ,u)

*is continuously Fréchet differentiable as a mapping*

$$X⟶L^{2}(0,T;W^{2,\infty }(\Omega ;R^{2})),$$

*and assume that the corresponding Liouville solutions are uniformly bounded in*

$$C([0,T];H^{2}(\Omega )).$$

*Then, Λ is Fréchet differentiable as a mapping*

$$\Lambda :O⟶C([0,T];L^{2}(\Omega )).$$

*For h=(δθ,δu)∈X, the derivative*

DΛ(θ,u)[h]=z

*is the unique solution of the linearized Liouville equation*

(3.8) $$\begin{array}{rlrl} \partial_{t}z+\nabla \cdot (bz) & =-\nabla \cdot (\delta b\;p), & & (x,t)\in \Omega \times (0,T),\\ z(x,0) & =0,\;x\in \Omega , \end{array}$$

*subject to the homogeneous boundary condition*

z=0on∂Ω×(0,T],

*where*

(3.9) $$\delta b=D_{\theta }b(\theta ,u)[\delta \theta ]+D_{u}b(\theta ,u)[\delta u].$$

Proof.
Fix v=(θ,u)∈O and let h=(δθ,δu)∈X be sufficiently small so that v+h∈O. Denote

$$p=\Lambda (v),\;p_{h}=\Lambda (v+h),\;b=b(v)\;\text{and}\;b_{h}=b(v+h).$$

Since the velocity field is Fréchet differentiable, we have

(3.10) $$b_{h}=b+\delta b+\rho_{b}(h),$$

where δb=Db(v)[h] and

(3.11) $$\frac{\|\rho_{b}(h){\|}_{L^{2}(0,T;W^{2,\infty }(\Omega ))}}{\|h{\|}_{X}}⟶0\;\text{as }\|h{\|}_{X}⟶0.$$

Let z be the solution of the linearized equation (3.8). We must show that

$$p_{h}-p-z=o(\|h{\|}_{X})$$

in $C([0,T];L^{2}(\Omega ))$. First set $q=p_{h}-p$. Subtracting the equations for $p_{h}$ and p gives

$$\partial_{t}q+\nabla \cdot (bq)=-\nabla \cdot ((b_{h}-b)p_{h})\;\text{and}\;q(x,0)=0.$$

Under the stated $H^{2}$ regularity assumptions, applying the same energy argument to the equation and to its first spatial derivatives gives

(3.12) $$\|q{\|}_{C([0,T];H^{1}(\Omega ))}\leq C\|b_{h}-b{\|}_{L^{2}(0,T;W^{2,\infty }(\Omega ))}.$$

By the Fréchet differentiability of b,

$$\|b_{h}-b{\|}_{L^{2}(0,T;W^{2,\infty }(\Omega ))}=O(\|h{\|}_{X}),$$

and therefore

(3.13) $$\|p_{h}-p{\|}_{C([0,T];H^{1}(\Omega ))}=O(\|h{\|}_{X}).$$

Now, we define the remainder

$$r=p_{h}-p-z.$$

Using the equations for $p_{h}$, p and z, together with equation (3.10), one obtains

(3.14) $$\partial_{t}r+\nabla \cdot (br)=-\nabla \cdot \left(\delta b\;(p_{h}-p)\right)-\nabla \cdot \left(\rho_{b}(h)p_{h}\right),$$

with

r(x,0)=0

and homogeneous boundary conditions. Applying the $L^{2}$ energy estimate used in proposition 8 to equation (3.14) gives

$$\begin{array}{r} \|r{\|}_{C([0,T];L^{2}(\Omega ))}\leq C(\|\delta b{\|}_{L^{2}(0,T;W^{1,\infty }(\Omega ))}\;\|p_{h}-p{\|}_{C([0,T];H^{1}(\Omega ))}\\ +\|\rho_{b}(h){\|}_{L^{2}(0,T;W^{1,\infty }(\Omega ))}\;\|p_{h}{\|}_{C([0,T];H^{1}(\Omega ))}). \end{array}$$

Since Db(v) is bounded,

$$\|\delta b{\|}_{L^{2}(0,T;W^{1,\infty }(\Omega ))}\leq C\|h{\|}_{X}.$$

Together with equation (3.13), the first term on the right-hand side is therefore

$$O(\|h{\|}_{X}^{2}).$$

Moreover, the family $p_{h}$ is uniformly bounded in $C([0,T];H^{1}(\Omega ))$, while equation (3.11) implies

$$\|\rho_{b}(h){\|}_{L^{2}(0,T;W^{1,\infty }(\Omega ))}=o(\|h{\|}_{X}).$$

Consequently,

$$\|r{\|}_{C([0,T];L^{2}(\Omega ))}=o(\|h{\|}_{X}).$$

Hence,

$$\frac{\|\Lambda (v+h)-\Lambda (v)-D\Lambda (v)[h]{\|}_{C([0,T];L^{2}(\Omega ))}}{\|h{\|}_{X}}⟶0$$

as $\|h{\|}_{X}\to 0$. Therefore, Λ is Fréchet differentiable from X into $C([0,T];L^{2}(\Omega ))$, with derivative characterized by equation (3.8).

Propositions 8 and 9 establish the continuous and Fréchet differentiable dependence of the Liouville state on the model parameters and controls in the spaces specified above. In the following proposition, we analyse important properties of the cost functionals $J_{1}$ and $J_{2}$ introduced in equations (2.17) and (2.18).

**Proposition 10.**

*The objective functionals $J_{1},J_{2}$, given in equations (2.17) and (2.18), are sequentially weakly lower semicontinuous (w.l.s.c.), bounded from below, coercive on $T_{\text{ad}},U_{\text{ad}}$, respectively.*

Proof.
The properties of being bounded from below and coercive for $J_{i}$ can be established using the fact that $J_{1},J_{2}$ are non-negative. To prove the weak lower semicontinuity (w.l.s.c.) property, we first observe that $J_{1}$ and $J_{2}$ are not convex in θ and u, respectively. However, the sets $T_{\text{ad}}$ and $U_{\text{ad}}$ are convex, closed and weakly sequentially compact. Specifically, if sequences $\left\{y^{n}\right\}$ and $\left\{z^{n}\right\}$ belong to $T_{\text{ad}}$ and $U_{\text{ad}}$ such that

$$\|y^{n}{\|}_{l^{2}}\leq D_{1}\;\text{and}\;\|z^{n}{\|}_{L^{2}}\leq D_{2},\;\text{for all }n,$$

then they are bounded. Consequently, there exist subsequences $\left\{y^{n_{k}}\right\}$ and $\left\{z^{n_{k}}\right\}$ such that

$$y^{n_{k}}⇀y\in T_{\text{ad}}\;\text{and}\;z^{n_{k}}⇀z\in U_{\text{ad}}.$$

In addition, it follows that

$$\|y{\|}_{l^{2}}\leq \text{lim inf }\|y^{n}{\|}_{L^{2}}\leq D_{1}\;\text{and}\;\|z{\|}_{L^{2}}\leq \text{lim inf }\|z^{n}{\|}_{L^{2}}\leq D_{2},$$

ensuring that $y\in U_{\text{ad}}$ and $z\in V_{\text{ad}}$. Next, we define the sets

$$T_{\eta_{1}}=\{\theta \in T_{\text{ad}}:J_{1}(\theta )\leq \eta_{1}\}\;\text{and}\;V_{\eta_{2}}=\{u\in U_{\text{ad}}:J_{2}(u)\leq \eta_{2}\}.$$

Since $J_{i}$ is continuous, the sets $T_{\eta_{1}}$ and $V_{\eta_{2}}$ are closed for all $\eta_{i}\in R$. As they are closed subsets of the weakly sequentially compact spaces $U_{\text{ad}}$ and $V_{\text{ad}}$, they are also weakly sequentially closed for all $\eta_{i}\in R$. This confirms that $J_{1}$ and $J_{2}$ are weakly sequentially lower semicontinuous.

We now state and prove the existence of an optimal parameter set $\theta^{*}$ and the corresponding optimal drug dosage concentrations $u^{*}$ in the following theorem.

**Theorem 3.1.**

*Let $p_{0}\in H^{1}(\Omega )$, and let the cost functionals $J_{1}$ and $J_{2}$ be defined as in equations (2.17) and (2.18), respectively. Then, there exist pairs $(p_{1}^{*},\theta^{*})\in C([0,T];H^{1}(\Omega ))\times T_{\text{ad}}$ and $(p_{2}^{*},u^{*})\in C([0,T];H^{1}(\Omega ))\times U_{\text{ad}}$ such that $p_{1}^{*}$ and $p_{2}^{*}$ are solutions of $E(p_{0},\theta^{*},0)=0$ and $E(p_{0},\theta^{*},u^{*})=0$, respectively, and $\theta^{*}$ and $u^{*}$ minimize $J_{1}$ and $J_{2}$ over $T_{\text{ad}}$ and $U_{\text{ad}}$, respectively.*

Proof.
We begin by proving the existence of a minimizer for $J_{1}$ in equation (2.17). Since $J_{1}$ is bounded below, there exists a minimizing sequence $(\theta^{m})\subset T_{\text{ad}}$. Given that $T_{\text{ad}}\subset R^{4}$ and that $J_{1}$ is coercive over $T_{\text{ad}}$, the sequence $\left(\theta^{m}\right)$ is bounded and therefore admits a convergent subsequence $\left(\theta^{m_{l}}\right)$ such that $\theta^{m_{l}}\to \theta^{*}\in T_{ad}$. Let $p^{m_{l}}:=\Lambda (\theta^{m_{l}},0)$. By the stability estimate equation (3.2), the sequence $\left(p^{m_{l}}\right)$ is bounded in $L^{2}(0,T;H^{1}(\Omega ))$. Moreover, the corresponding time derivatives $\left(\partial_{t}p^{m_{l}}\right)$ are bounded in $L^{2}(0,T;H^{1}(\Omega ))$, implying that, up to a subsequence, $p^{m_{l}}⇀p_{1}^{*}$ and $\partial_{t}p^{m_{l}}⇀\partial_{t}p_{1}^{*}$ weakly. It follows that the flux terms $\left(b(\theta^{m_{l}},0)p^{m_{l}}\right)$ also converge weakly in $L^{2}(0,T;L^{2}(\Omega ))$. This ensures that $p_{1}^{*}=\Lambda (\theta^{*},0)$, and since $J_{1}$ is weakly lower semicontinuous, the pair $\left(p_{1}^{*},\theta^{*}\right)$ minimizes $J_{1}$.

To establish the existence of a minimizer for $J_{2}$ as defined in equation (2.18), we proceed similarly. Since $U_{ad}$ is a closed subset of a Hilbert space and $J_{2}$ is coercive over $U_{ad}$, there exists a minimizing sequence $(u^{m})\subset U_{ad}$ that admits a weakly convergent subsequence $(u^{m_{l}})\to u^{*}\in U_{ad}$. Defining $p^{m_{l}}:=\Lambda (\theta^{*},u^{m_{l}})$, we again invoke the stability estimate and compactness arguments. By the Aubin–Lions lemma [40], a subsequence of $\left(p^{m_{l}}\right)$ converges strongly in $L^{2}(0,T;L^{2}(\Omega ))$, and weakly in $L^{2}(0,T;H^{1}(\Omega ))$, to some limit $p_{2}^{*}$. It follows that $p_{2}^{*}=\Lambda (\theta^{*},u^{*})$, and by the weak lower semicontinuity of $J_{2}$, the pair $\left(p_{2}^{*},u^{*}\right)$ minimizes $J_{2}$.

## 4. Numerical schemes for solving the optimization problems

In this section, we present the numerical discretization schemes used to solve the optimization problems equations (2.17) and (2.18). To solve for the forward equation (2.15) and adjoint Liouville equations (4.1) and (4.7), we use a strong stability preserving Runge–Kutta time discretization scheme coupled with a Kurganov–Tadmor spatial discretization scheme and also implement a Strang splitting method. The details of the complete scheme can be found in [41]. To solve for the optimal parameter set and optimal dosages, we describe the PMP-based SQH scheme in the sections below.

### 4.1. Adjoint-based parameter estimation and sequential quadratic Hamiltonian optimal control

We employ two related but distinct optimization strategies for the parameter-estimation and treatment-control problems. The unknown model parameters θ form a finite-dimensional vector and are estimated using a Lagrangian adjoint formulation together with a projected nonlinear conjugate-gradient (NCG) method. By contrast, the treatment variables $u=(u_{1},u_{2})$ are time-dependent controls, and the corresponding optimal control problem is solved using the SQH framework based on the PMP.

#### 4.1.1. Adjoint-based estimation of ${}^{*}$

For a fixed treatment control u=0, the parameter-estimation problem equation (2.17) is a finite-dimensional optimization problem constrained by the Liouville equation. Let p=Λ(θ,0) denote the solution of

$$E(p_{0},\theta ,0)=0.$$

The reduced objective functional is defined by

$${\hat{J}}_{1}(\theta )=J_{1}(\theta ,\Lambda (\theta ,0)),$$

so that the parameter-estimation problem can equivalently be written as

$$\min_{\theta \in T_{\text{ad}}}{\hat{J}}_{1}(\theta ).$$

We introduce the Lagrangian

$$L(p,\theta ,q)=J_{1}(\theta ,p)+\int_{0}^{T}\int_{\Omega }q\left[\partial_{t}p+\nabla \cdot (b(x,\theta ,0)p)\right]\;\text{d}x\;\text{d}t,$$

where q is the adjoint variable. Taking the first variation of L with respect to the state p and choosing q so that the terms involving the state variation vanish yields the adjoint equation

(4.1) $$\begin{array}{rl} -\partial_{t}q(x,t)-b(x,\theta ,0)\cdot \nabla q(x,t) & =\frac{\alpha }{p(x,t)},\;\text{in}\;\Omega \times (0,T),\\ q(x,T) & =0,\;\;\text{in}\;\Omega , \end{array}$$

where p satisfies

$$E(p_{0},\theta ,0)=0.$$

Once the forward state p and adjoint state q have been computed, the derivative of the reduced objective with respect to the jth component of θ can be evaluated without explicitly computing $\partial p/\partial T_{j}$. In particular,

(4.2) $$\frac{\partial {\hat{J}}_{1}}{\partial T_{j}}=\beta T_{j}-\int_{0}^{T}\int_{\Omega }\left(\frac{\partial b}{\partial T_{j}}\cdot \nabla q\right)p\;\text{d}x\;\text{d}t,\;j=1,\ldots ,4.$$

Thus, the gradient of the reduced objective is obtained from one forward Liouville solve and one adjoint solve.

The estimated parameter vector is subsequently obtained using a projected NCG algorithm [26,42–44]. Given an initial estimate $\theta^{0}\in T_{\text{ad}}$, the initial search direction is

$$d^{0}=-\nabla {\hat{J}}_{1}(\theta^{0}).$$

At iteration k, a line search is used to determine the step length $\alpha_{k}$, and the parameter vector is updated according to

(4.3) $$\theta^{k+1}=P_{T_{\text{ad}}}\left(\theta^{k}+\alpha_{k}d^{k}\right),$$

where $P_{T_{\text{ad}}}$ denotes the projection onto the admissible parameter set. The new conjugate direction is constructed as

(4.4) $$d^{k+1}=-\nabla {\hat{J}}_{1}(\theta^{k+1})+\eta_{k}d^{k},$$

where the Dai–Yuan coefficient used in the numerical implementation is

(4.5) $$\eta_{k}=\frac{\|\nabla {\hat{J}}_{1}(\theta^{k+1}){\|}_{l^{2}}^{2}}{(d^{k}{)}^{T}\left[\nabla {\hat{J}}_{1}(\theta^{k+1})-\nabla {\hat{J}}_{1}(\theta^{k})\right]}.$$

The forward and adjoint equations are recomputed following each parameter update, and the procedure is continued until the gradient norm satisfies the prescribed stopping criterion.

For a better global parameter estimate, the optimization is initialized from multiple points distributed throughout $T_{\text{ad}}$. The projected NCG algorithm is applied independently to each initial estimate, and the parameter vector yielding the smallest value of the objective functional is selected as the final estimate $\theta^{*}$.

#### 4.1.2. Sequential quadratic Hamiltonian framework for estimating $u^{*}$

Once the parameter vector $\theta^{*}$ has been identified, we solve the time-dependent treatment optimization problem equation (2.18). This sequential formulation ensures that the underlying tumour–immune dynamics is first calibrated from the population data before therapeutic intervention is optimized, thereby separating the identification of the patient-population dynamics from the subsequent design of treatment. By contrast with the finite-dimensional parameter-estimation problem, the chemotherapy and immunotherapy dosages $u=(u_{1},u_{2})$ are time-dependent variables belonging to the infinite-dimensional admissible control space $U_{\text{ad}}$. We therefore formulate the corresponding optimality conditions using the PMP and solve the resulting optimal control problem numerically using the SQH method.

From a biological perspective, the objective is to determine time-dependent chemotherapy and immunotherapy dosages that drive the population distribution of cancer and immune cells towards a desired therapeutic state. At the same time, the control-dependent penalty terms discourage excessive drug administration. The optimal control formulation therefore balances the desired modification of the tumour–immune response with the need to limit unnecessary treatment exposure, providing a biologically meaningful framework for designing treatment strategies at the population level.

For the treatment-control problem, we construct the Hamiltonian–Pontryagin functions

(4.6) $$\begin{array}{rl} H_{1}(t,u_{1}(t),p(\cdot ,t),q(\cdot ,t)) & =\frac{\gamma_{1}}{2}u_{1}^{2}(t)+\beta_{1}|u_{1}(t)|-\int_{\Omega }(b(u)\cdot \nabla q(x,t))p(x,t)\;\text{d}x\\ \text{and }H_{2}(t,u_{2}(t),p(\cdot ,t),q(\cdot ,t)) & =\frac{\gamma_{2}}{2}u_{2}^{2}(t)+\beta_{2}|u_{2}(t)|-\int_{\Omega }(b(u)\cdot \nabla q(x,t))p(x,t)\;\text{d}x. \end{array}$$

Here, q solves the adjoint equation

(4.7) $$\begin{array}{rlrl} -\partial_{t}q(x,t)-b(x,\theta^{*},u)\cdot \nabla q(x,t) & =-\nu (p(x,t)-p^{d}(x,t)), & & \text{in }\Omega \times (0,T),\\ q(x,T) & =0,\;\text{in }\Omega , \end{array}$$

with p satisfying

$$E(p_{0},\theta^{*},u)=0.$$

The Pontryagin minimum condition gives the following characterization of an optimal treatment control.

**Theorem 4.1.**

*Let $u^{*}$ be a solution of equation (2.18). Then,*

$$H_{i}(t,u_{i}^{*}(t),p^{*}(\cdot ,t),q^{*}(\cdot ,t))=\min_{v\in [0,D_{i}]}H_{i}(t,v,p^{*}(\cdot ,t),q^{*}(\cdot ,t)),\;i=1,2,$$

*for almost every t∈(0,T), where $p^{*}$ and $q^{*}$ solve equations (2.15) and (4.7), respectively, with the optimal control $u^{*}$.*

To numerically determine $u^{*}$, we employ the SQH method. At each iteration, the forward Liouville equation and the corresponding adjoint equation are solved for the current control. The Hamiltonian is then minimized pointwise with respect to the admissible control values, with a quadratic stabilization term introduced between successive iterates. This sequential Hamiltonian minimization provides an updated admissible control while avoiding the explicit computation of derivatives of the Hamiltonian with respect to u. The procedure is repeated until the change in the objective functional and control satisfies the prescribed convergence criterion.

Thus, the overall computational framework consists of two stages: an adjoint-based projected NCG method for identifying the finite-dimensional parameter vector $\theta^{*}$, followed by an SQH method for determining the optimal time-dependent treatment control $u^{*}$.

### 4.2. A sequential quadratic Hamiltonian method

The SQH method, introduced in [45], represents a recent advancement in the class of successive approximation techniques, which have their origins in foundational works such as [46–49]. These methods are based on iteratively minimizing the Hamilton–Pontryagin (HP) function associated with a given optimal control problem. Initially inspired by needle variations in the proof of the PMP [50] and an early result by Rozonoer [51], this approach is well suited for addressing the optimal drug dosage determination problem described in equation (2.18).

To describe this method, we denote H(A,p,q) as the generic notation for all the three HP functions $H_{\theta },H_{1}\text{ and }H_{2}$ with the optimization variable A in the minimization of a functional J. The starting point of our SQH method is the augmented HP function, which is defined as

$$H_{\epsilon }(A,\tilde{A},p,q)=H(A,p,q)+\epsilon (A-\tilde{A}{)}^{2},$$

where ϵ>0 is a penalization parameter that is adaptively adjusted at each iteration of the SQH process. Specifically, ϵ is increased if a sufficient decrease in the functional J is not observed, and decreased if J decreases adequately. Here, $\tilde{A}$ represents the previous approximation of A. The purpose of the quadratic term $\epsilon (A-\tilde{A}{)}^{2}$ is to ensure that the pointwise minimizer of $H_{\epsilon }$, and thus the updates to A, remain close to the prior values $\tilde{A}$, especially when ϵ is large. Note that during each optimization step, the values of p and q used are those obtained from the previous iteration. The SQH algorithm is outlined in the following algorithm:

**Algorithm 4.1** (SQH method).

**Figure:**
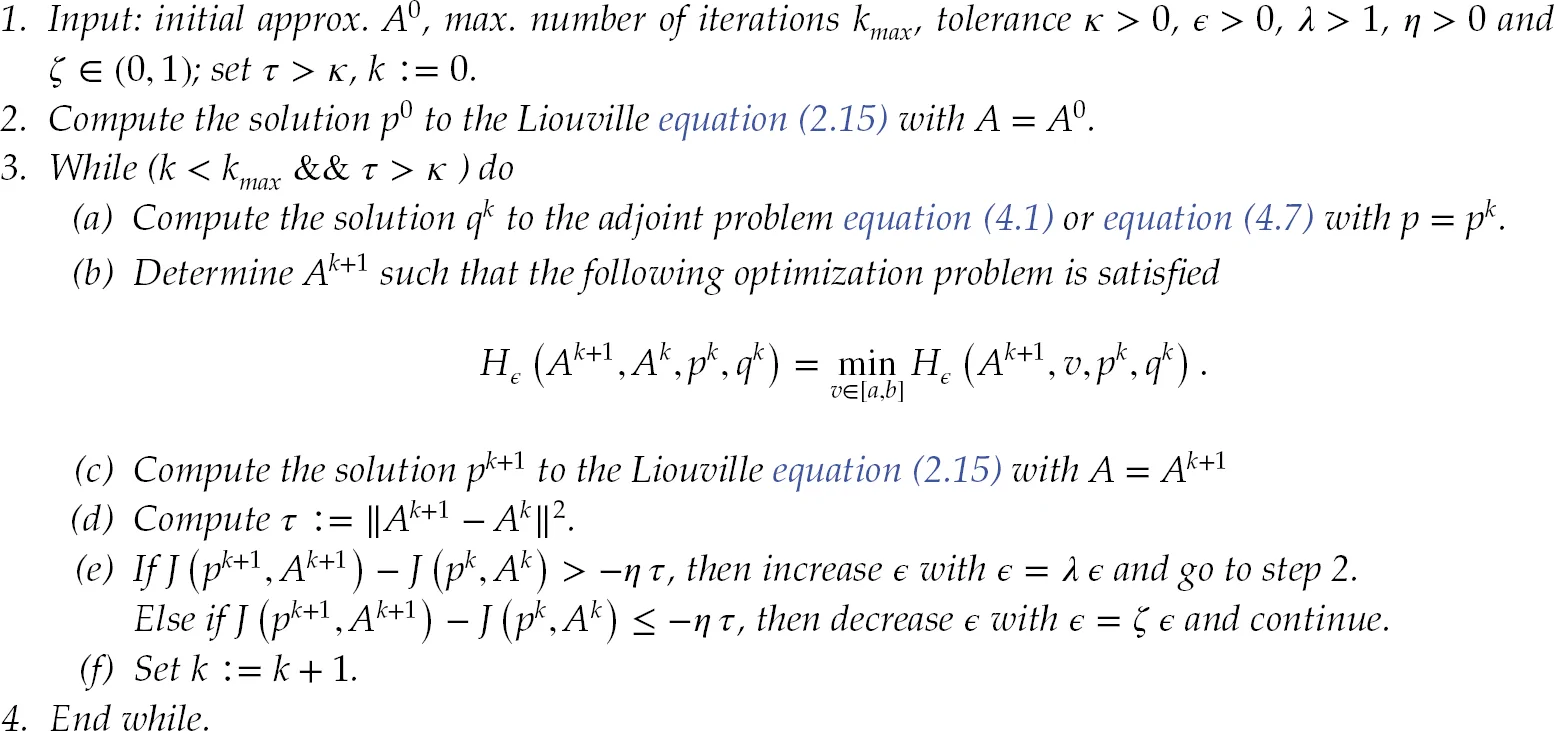

In step (e) of this algorithm, if the inequality $J\left(p^{k+1},A^{k+1}\right)-J\left(p^{k},A^{k}\right)>-\eta \;\tau$ holds, it indicates that a sufficient decrease in the objective functional J has not been achieved. In such a case, ϵ is increased (since λ>1), and the optimization in step (b) is repeated with the updated augmented HP function. Conversely, if the inequality does not hold, it confirms that the required reduction in J has been met. The updated $A^{k+1}$ is then adopted, along with the corresponding updates $p^{k+1}$ and $q^{k+1}$ for the Liouville and its adjoint states. In this situation, ϵ is reduced by a factor ζ<1.

Using arguments similar to those presented in [32], we now state the descent property of the SQH algorithm applied to the treatment optimal control problem equation (2.18).

**Theorem 4.2.**

*Let $\left(p^{k},u^{k}\right)\;and\;\left(p^{k+1},u^{k+1}\right)$ be successive iterates generated by the SQH method (algorithm 4.1) applied to equation (2.18), with $u^{k},u^{k+1}\in U_{\text{ad}}$. Then, there exists a constant θ>0, independent of ϵ and the current iterate $u^{k}$, such that, for the current value of ϵ>0 chosen by algorithm 4.1,*

(4.8) $$J_{2}\left(p^{k+1},u^{k+1}\right)-J_{2}\left(p^{k},u^{k}\right)\leq -\left(\epsilon -\theta \right){\left\|u^{k+1}-u^{k}\right\|}_{(L^{2}(0,T){)}^{2}}^{2}.$$

*Consequently, whenever ϵ>θ, an accepted SQH update produces a decrease in the objective functional $J_{2}$ unless*

$$u^{k+1}=u^{k}.$$

Theorem 4.2 provides the descent property underlying the adaptive choice of the penalization parameter ϵ in the SQH algorithm. If the decrease in $J_{2}$ is insufficient, ϵ is increased so that successive control iterates remain closer to one another. Once the sufficient-decrease condition is satisfied, the update is accepted and ϵ is reduced. Thus, the quadratic Hamiltonian regularization stabilizes the control updates while preserving descent of the objective functional.

## 5. Numerical results

In this section, we present numerical simulations that demonstrate the effectiveness of our Liouville framework. We use the non-dimensional scaling parameters $M_{1}$, $M_{2}$ and $M_{3}$, introduced in equation (2.2), with values $M_{1}=3\times 10^{-5}$, $M_{2}=2.25\times 10^{-7}$ and $M_{3}=10^{-2}$. We begin by addressing the parameter estimation problem described in equation (2.17). The spatial domain is defined as $\Omega =(0,5{)}^{2}$ and discretized using $N_{x_{i}}=51$ grid points along each spatial direction for i=1,2. The final simulation time is set to t=0.5 with a temporal resolution of $N_{t}=1001$ time steps. The synthetic population data are modelled as a set of target PDFs $p_{i}^{*}(x)$ for i=1,…,N, where N=3. In our numerical simulations, these target PDFs are constructed from synthetic data and are intended to represent a pseudo-clinical setting in which measurements of cancer burden and immune response are available only at a limited number of observation times, analogous to measurements obtained during clinical visits. Rather than representing repeated measurements from a single patient, each PDF describes population-level variability in the cancer and immune cell states across a heterogeneous patient cohort at a given observation time. Thus, the numerical experiments are designed to examine whether the Liouville framework can recover and propagate population-level information from sparse observations of a heterogeneous cohort. Each $p_{i}^{*}$ is assumed to follow a Gaussian distribution centred at the measured mean $E[p_{i}^{*}]$ and with a variance of 0.05. These data are interpolated in three dimensions to construct a continuous target function $p^{*}(x,t)$ over all discrete time points $t_{k}$, for $k=1,\ldots ,N_{t}$. The cost functional $J_{1}$ uses weights α=10 and β=0.01. The admissible parameter set $T_{\text{ad}}$ is defined with an upper bound vector $T=(T_{1},T_{2},T_{3},T_{4})=(7.0,5.0,5.0,5.0)$. The values of the non-estimated parameters are chosen as $k_{1}=4.2,\;d_{1}=6.6,\;d_{4}=1.2,\;L_{1}=10\text{ and}\;S_{1}=S_{2}=5.$ Since the numerical experiments are performed using synthetic data, these values are selected as representative reference values that generate biologically meaningful cancer–immune dynamics, including bounded tumour growth and a sustained immune response. The remaining parameters governing the cancer–immune interactions are treated as unknown and are estimated from the synthetic population data.

For prescribing the initial guesses in the parameter estimation problem, we note that the parameters are defined within specific intervals denoted by $\left[0,T_{i}\right]$. For each parameter, this interval is divided into four equally spaced points. Then, we combine these points across all parameters. Since there are four parameters in this case, the total number of combinations is 256. This represents the space of discrete combinations of the four parameters within their defined intervals, which are used as initial guesses for the parameter estimation problem. After obtaining the parameter θ using the NCG algorithm for each initial guess, we calculate the KL divergence between the PDF $p_{\theta }$ corresponding to the estimated parameter set θ and the target data $p^{*}$, defined as

$$\|p{\|}_{KL}=\int_{Q}p^{*}(x,t)\log\left(\frac{p^{*}(x,t)}{p_{\theta }(x,t)}\right)\;\text{d}x\text{d}t.$$

Finally, we choose the optimal parameter set θ that gives the least $\|p{\|}_{KL}$.

For the optimal drug dosage problem given in equation (2.18), the cost functional $J_{2}$ is defined using weights ν=50, $\gamma_{1}=0.01$, $\gamma_{2}=0.01$, $\beta_{1}=1$ and $\beta_{2}=5$. The parameters used for the SQH optimization algorithm are: σ=1.1, ζ=0.9, $\eta =1.0\times 10^{-5}$ and $\kappa =1.0\times 10^{-5}$, a maximum iteration count $k_{\max}=5000$, and an initial penalty parameter ϵ=1.0. The admissible control set $U_{\text{ad}}$ is defined with maximum drug dosages $D_{1}=1.0$ and $D_{2}=1.0$. The initial guesses for the control functions $u_{i}(t)$ are taken as constant functions with value 1.0.

### 5.1. Test case 1: weak immune system

In this test case, we generate synthetic measurement data to represent a hypothetical OC patient with a weakened immune response. To construct this dataset, we solve the ODE system equation (2.3) with initial condition (3,3) and a non-dimensional parameter vector θ=(6.41,1.0,2.5,3.0) over the time interval t∈[0,0.5], which corresponds to a physical time span of 50 days. The solution is sampled at N=3 distinct time points within this interval. Using the ODE solution values at these time points as the mean, we construct the corresponding target PDFs $p_{i}^{*}$, which serve as the synthetic measurement data for the parameter estimation problem.

Figure 1 presents contour plots comparing the PDFs of the actual data points $\left(y_{1},y_{2}\right)$ with those obtained from the Liouville-based parameter estimation. The top row shows the contour plots of the data-derived PDFs at times t=0, 0.25 and 0.5, where the true data point is marked with a dot. The bottom row displays the corresponding PDFs obtained from the Liouville framework at the same time instances, again indicating the true data point with a dot. We observe that the mean of the estimated PDF closely aligns with the actual data point, demonstrating that the Liouville framework accurately captures the mean behaviour of the random variables $\left(y_{1},y_{2}\right)$ while simultaneously retaining information about their population-level variability.

**Figure 1.**
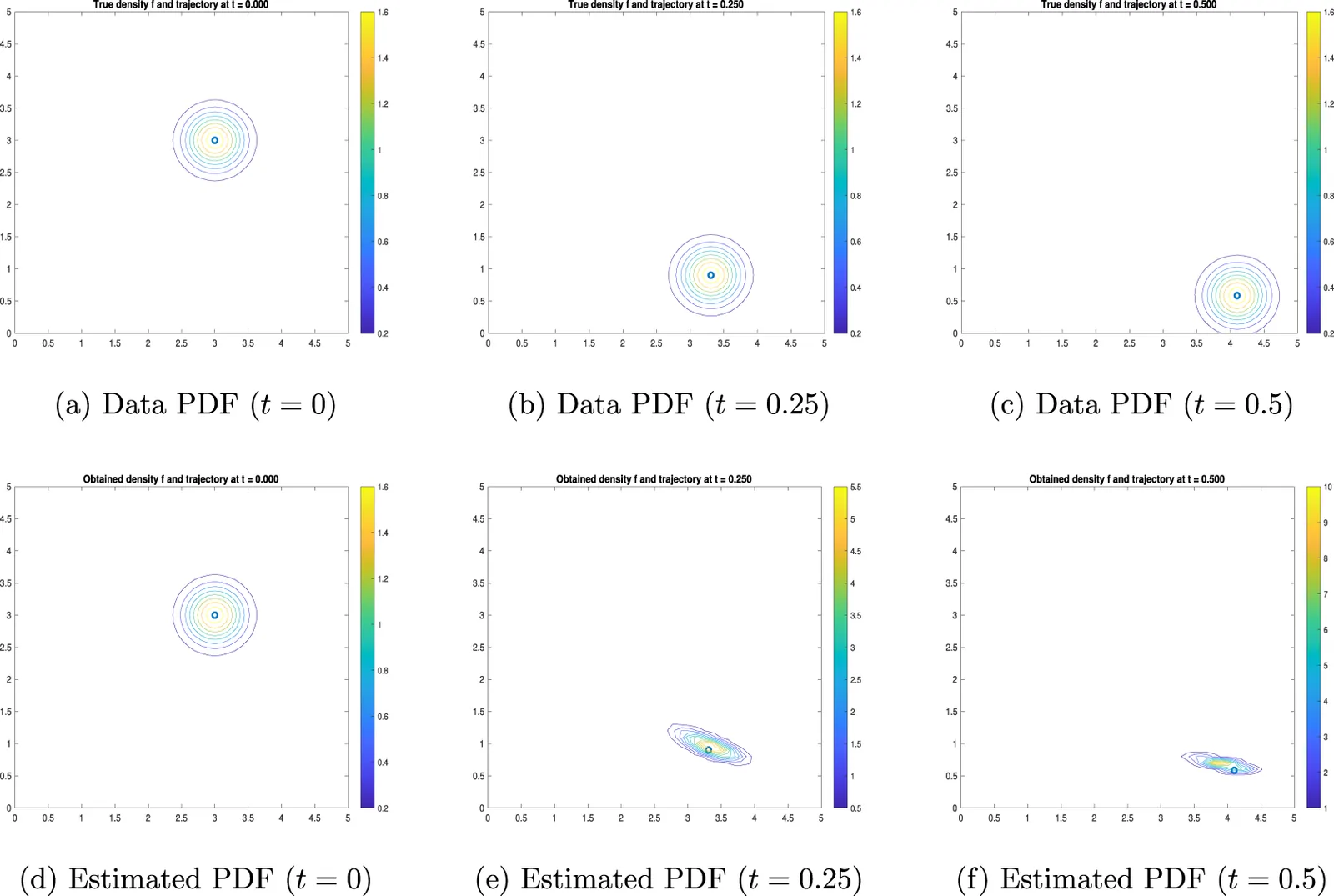
Test case 1: data fitting-plots of the PDF. (a) Data PDF (t=0), (b) data PDF (t=0.25), (c) data PDF (t=0.5), (d) estimated PDF (t=0), (e) estimated PDF (t=0.25) and (f) estimated PDF (t=0.5).

To further investigate the role of population heterogeneity in the parameter estimation problem, we perform an additional experiment associated with test case 1 in which the Liouville-based parameter estimation is compared directly with parameter estimation using the underlying deterministic ODE model. In particular, we retain the parameter vector θ=(6.41,1.0,2.5,3.0) and the initial mean state (3,3) used above, but replace the point initial condition by a Gaussian probability density centred at (3,3). The standard deviation $\sigma_{0}$ of this initial density is varied as $\sigma_{0}=0.1,0.3$ and 0.6 to represent increasing levels of population heterogeneity. The resulting initial density is propagated using the Liouville equation to generate the synthetic population data. The deterministic ODE parameters are estimated using the population means at the measurement times and a fixed initial condition (3,3), whereas the Liouville-based estimation uses the corresponding probability distributions.

Table 2 lists a comparison the two approaches in terms of relative parameter error, population KL error and mean-state RMSE. For small and moderate initial variability, the deterministic ODE model provides a competitive approximation of the mean population behaviour, although the Liouville formulation gives a smaller relative parameter error. The distinction becomes more pronounced for the broader initial distribution. In particular, for $\sigma_{0}=0.6$, the relative parameter error decreases from 0.421 for the ODE approach to 0.268 for the Liouville approach, while the population KL error decreases from 1.202 to 0.410. The mean-state RMSE similarly decreases from 0.144 to 0.039. These results illustrate that when the population is narrowly concentrated, fitting a deterministic ODE trajectory to population-level observations can provide a reasonable approximation, whereas explicitly propagating the probability distribution becomes advantageous as the heterogeneity of the initial population increases.

**Table 2.** Comparison of deterministic ODE and Liouville-based parameter estimation for test case 1 under different levels of initial population heterogeneity.

	relative parameter error		population KL error		mean-state RMSE	
$\sigma_{0}$	ODE	Liouville	ODE	Liouville	ODE	Liouville
0.1	0.342	0.244	3.416	4.113	0.074	0.146
0.3	0.344	0.290	1.993	2.137	0.076	0.417
0.6	0.421	0.268	1.202	0.410	0.144	0.039

Next, we address the problem of computing optimal dosages of chemotherapeutic and immunotherapeutic drugs, denoted by $u_{1}(t)$ and $u_{2}(t)$, respectively. We begin with the state $\left(y_{1},y_{2}\right)$ at the final time t=0.5 (non-dimensional units), as estimated in figure 1, representing a diagnosed case of OC. The goal is to determine optimal control input over a future non-dimensional time interval t∈[0,0.21], corresponding to 21 actual days, to transition the patient towards a cancer-free state.

To model the desired outcome, we construct a target PDF $p^{d}(x,t)$ by solving the Liouville equation (2.15) with zero drug input (u=0) and a modified parameter vector θ=(6.41,3.0,2.5,3.0), which reflects a higher cancer-cell death rate and thus represents a controlled or cured state. Finally, we solve the optimal control problem defined in equation (2.18) to compute the optimal drug dosage profiles that achieve this transition.

Figure 2 illustrates the evolution of the mean PDFs for three scenarios: the desired outcome, the trajectory without treatment and the trajectory with optimized treatment. In figure 3, we present the corresponding control profiles and the associated mean trajectories, both in the absence and presence of treatment. In the untreated case, the mean PDF follows a trajectory indicative of disease progression towards an uncontrolled cancer stage, characterized by aggressive tumour growth. By contrast, with the administration of the optimized combination of chemotherapeutic and immunotherapeutic agents, the mean PDF is effectively guided towards the desired controlled state, reflecting therapeutic success. It is also evident from the control profiles that achieving the targeted cancer-free state necessitates relatively high dosages of both drugs over the treatment period.

**Figure 2.**
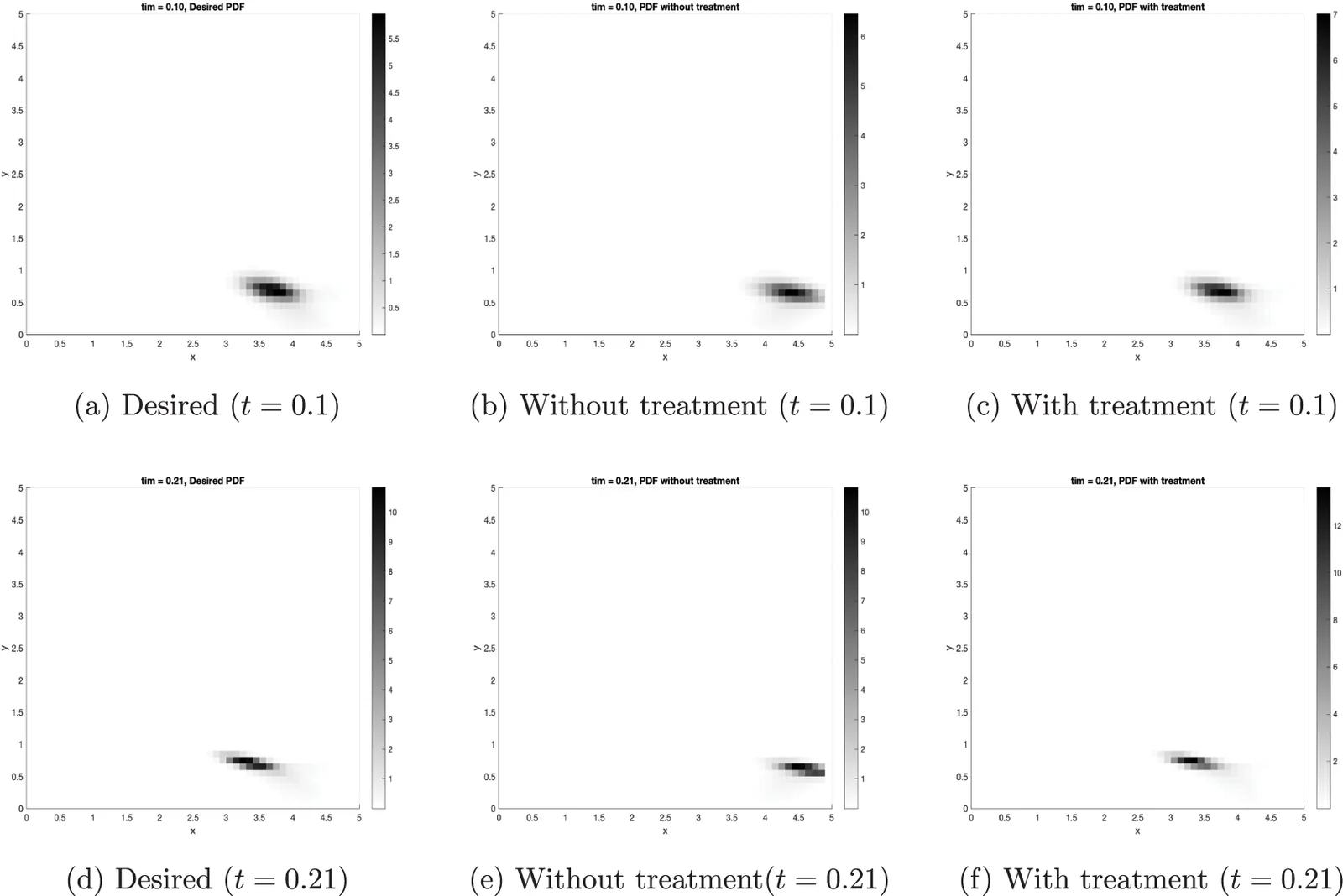
Test case 1: plots of the PDF without and with treatment. (a) Desired (t=0.1), (b) without treatment (t=0.1), (c) with treatment (t=0.1), (d) desired (t=0.21), (e) without treatment(t=0.21) and (f) with treatment (t=0.21).

**Figure 3.**
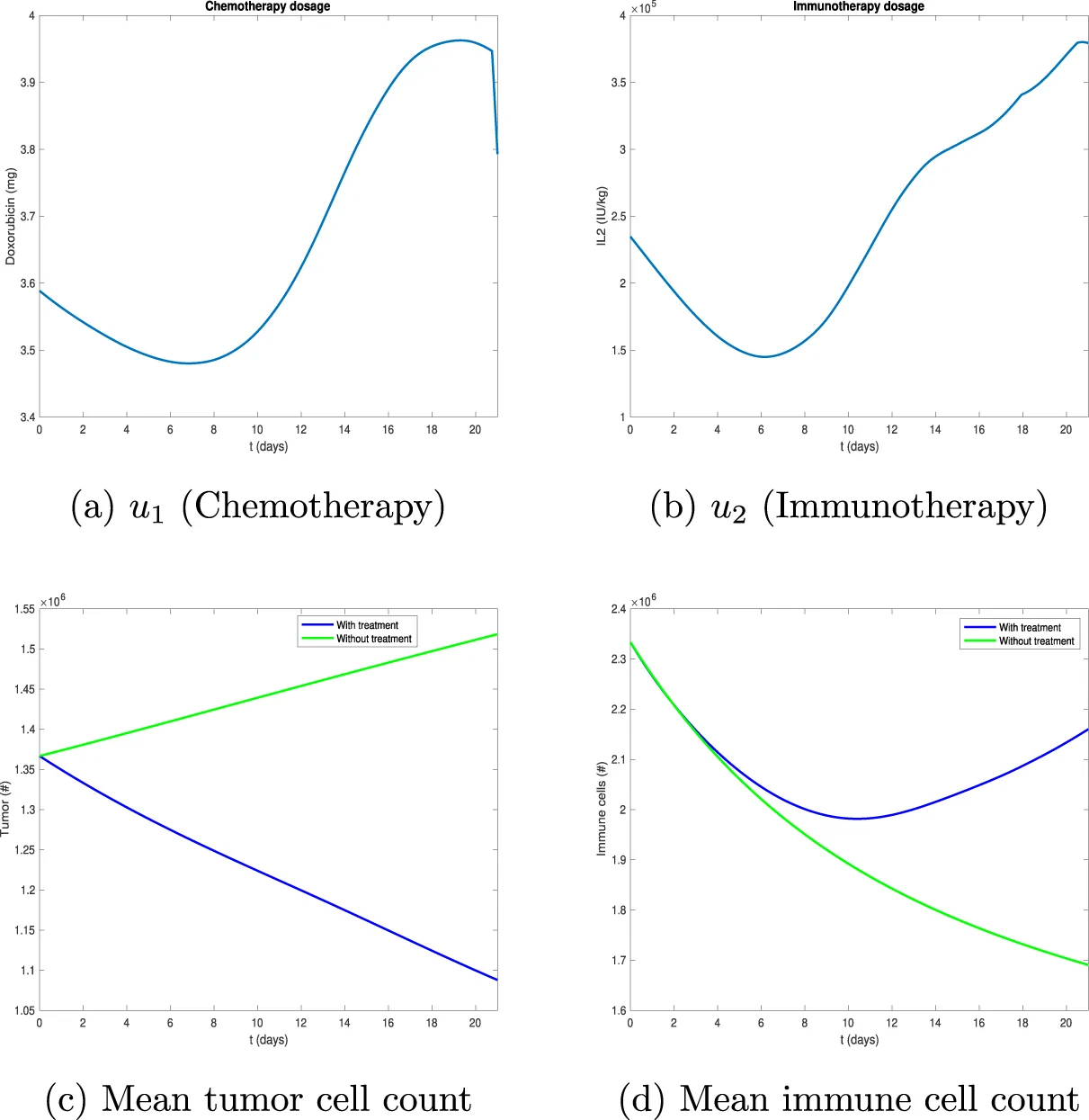
Test case 1: plots of the therapies. (a) $u_{1}$ (chemotherapy), (b) $u_{2}$ (immunotherapy), (c) mean tumour cell count and (d) mean immune cell count.

### 5.2. Test case 2: moderate immune system

In this test case, we generate synthetic measurement data to represent a hypothetical OC patient with a moderately responsive immune system. To construct this dataset, we solve the ODE system equation (2.3) with the initial condition (3,3) and a non-dimensional parameter set θ=(6.41,1.8,2.5,3.0) over the time interval t∈[0,0.5], corresponding to a physical duration of 50 days. The solution is sampled at N=3 time points within this interval. Using the ODE solution values at these time points as the mean, we construct the target PDFs $p_{i}^{*}$, which serve as synthetic measurement data for the parameter estimation process.

Figure 4 displays the contour plots of the PDFs corresponding to the actual data points $\left(y_{1},y_{2}\right)$ and the Liouville-based fits. The top row shows the PDFs of the measured data at time points t=0, 0.25 and 0.5, with the actual data point indicated by a dot. The bottom row presents the corresponding Liouville PDF estimates, with the true data point again marked. As observed previously, the mean of the estimated PDF closely aligns with the measured data, underscoring the capability of the Liouville framework to provide accurate reconstructions. These results further highlight the flexibility of our approach in capturing the dynamics of patient subgroups with varying characteristics.

**Figure 4.**
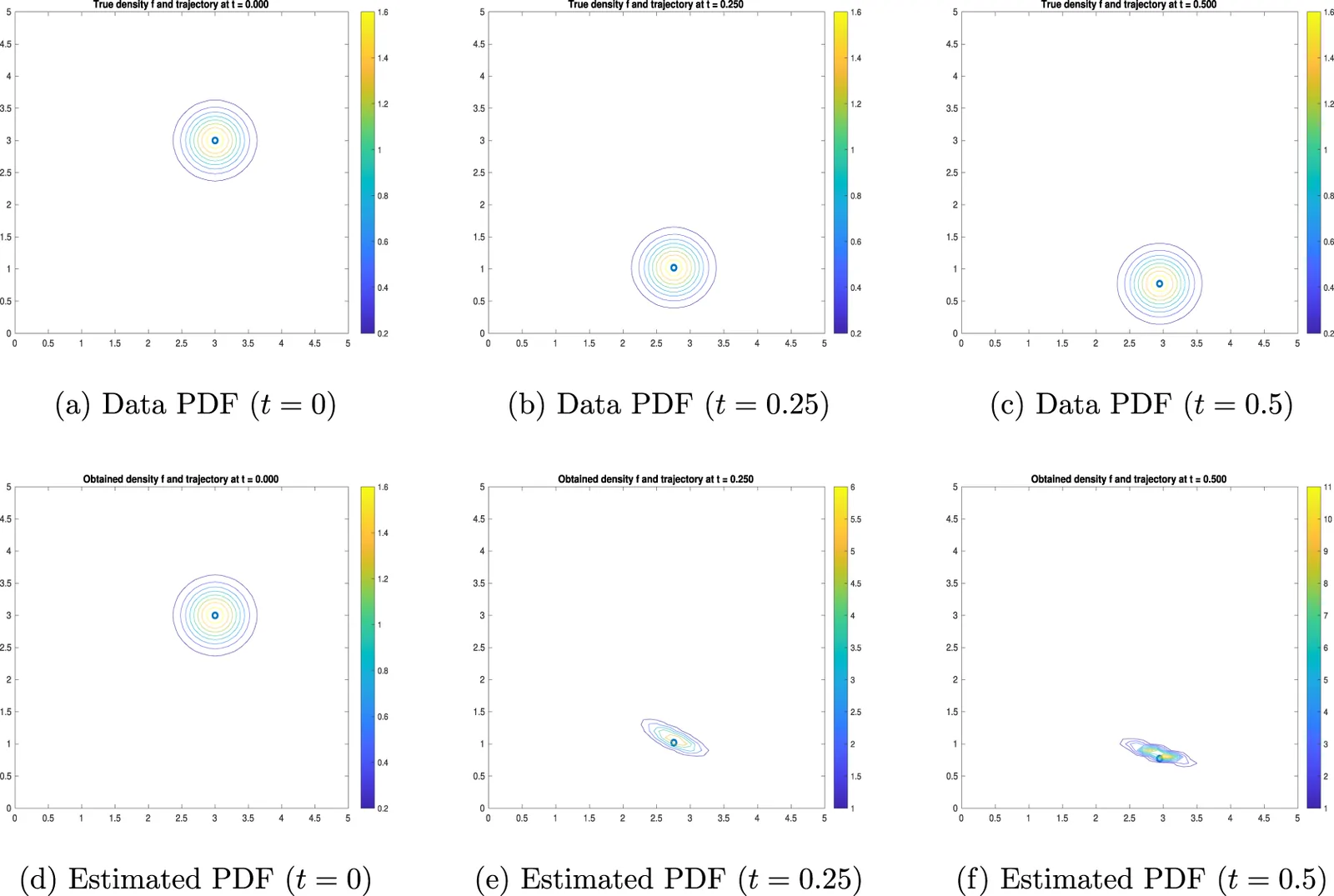
Test case 2: data fitting-plots of the PDF. (a) Data PDF (t=0), (b) data PDF (t=0.25), (c) data PDF (t=0.5), (d) estimated PDF (t=0) (e) estimated PDF (t=0.25) and (f) estimated PDF (t=0.5).

We then proceed to compute the optimal dosages of the chemotherapeutic and immunotherapeutic drugs, denoted by $u_{1}(t)$ and $u_{2}(t)$, respectively. The initial condition for this optimization is taken as the state $\left(y_{1},y_{2}\right)$ at the final time t=0.5 (non-dimensional) from figure 4, representing a diagnosed case of OC. Our objective is to determine the optimal control input over the subsequent non-dimensional interval t∈[0,0.21], equivalent to 21 days, so as to drive the system towards a desired cancer-free state. As in test case 1, the target is modelled using the same desired PDF $p^{d}(x,t)$, obtained from the Liouville system with zero control input and modified parameters indicating a higher cancer-cell death rate. We solve the optimal control problem defined in equation (2.18) to compute the optimal dosage profiles.

Figure 5 illustrates the evolution of the mean PDFs under three scenarios: the desired outcome, the trajectory without treatment and the trajectory with optimal treatment. Figure 6 presents the corresponding control input and the associated mean trajectories, both in the absence and presence of treatment. In the absence of therapy, the system evolves along a trajectory indicative of an uncontrolled cancer stage, characterized by aggressive tumour progression. However, upon administering the optimized combination of chemotherapeutic and immunotherapeutic agents, the mean PDF is successfully steered towards the desired controlled state. Notably, the control plots reveal a reduced dosage requirement for both drugs compared to the previous test case. The chemotherapy drug is administered at the beginning of the treatment period, with a gradual tapering towards the final time. By contrast, the immunotherapy drug is applied briefly around day 15 and then turned off, demonstrating a sparsity pattern in its administration. This behaviour is intuitive, as the patient subgroup in this test case exhibits a stronger immune response, thereby requiring less aggressive intervention to achieve the same therapeutic outcome.

**Figure 5.**
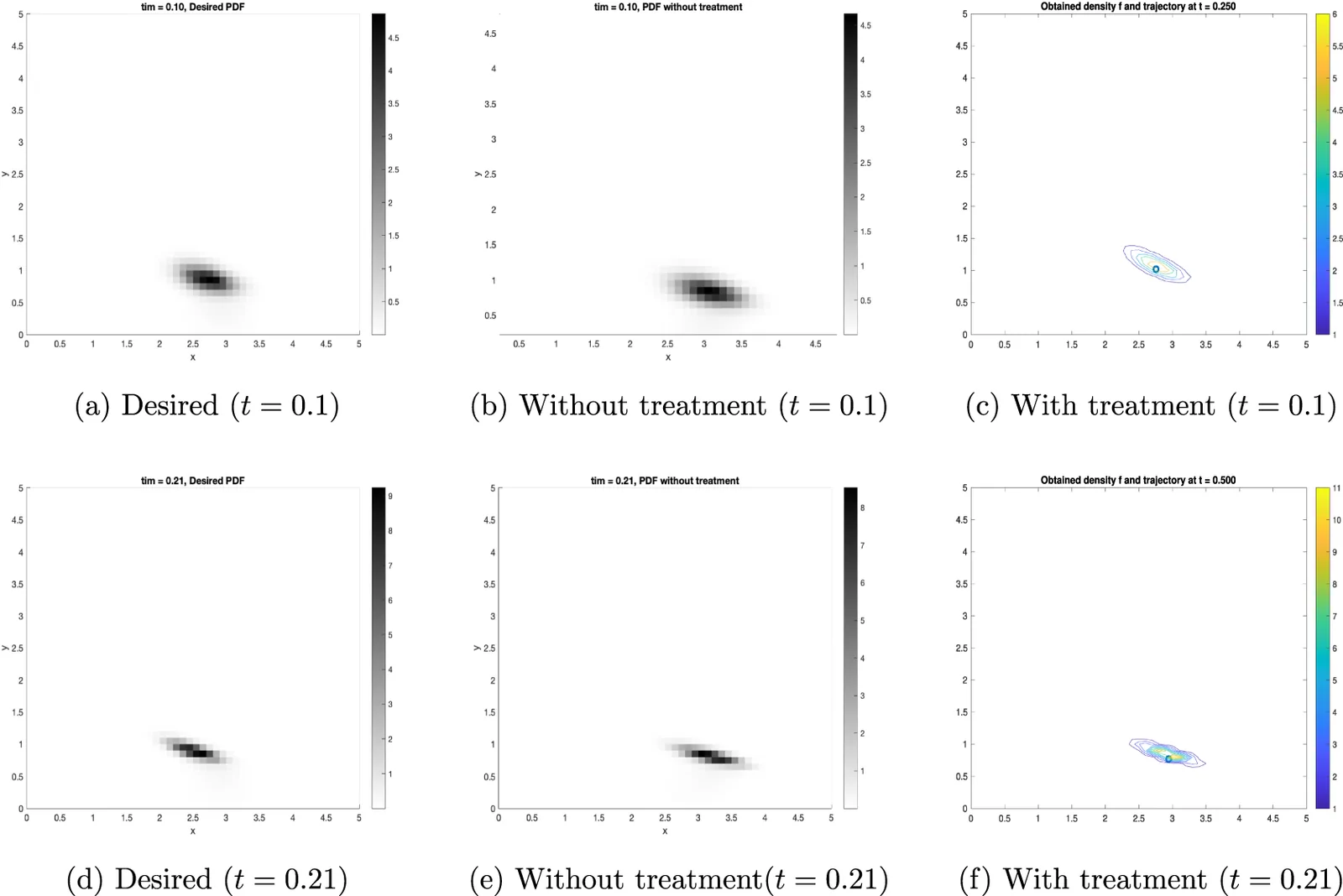
Test case 2: plots of the PDF without and with treatment. (a) Desired (t=0.1), (b) without treatment (t=0.1), (c) with treatment (t=0.1), (d) desired (t=0.21), (e) without treatment(t=0.21) and (f) with treatment (t=0.21).

**Figure 6.**
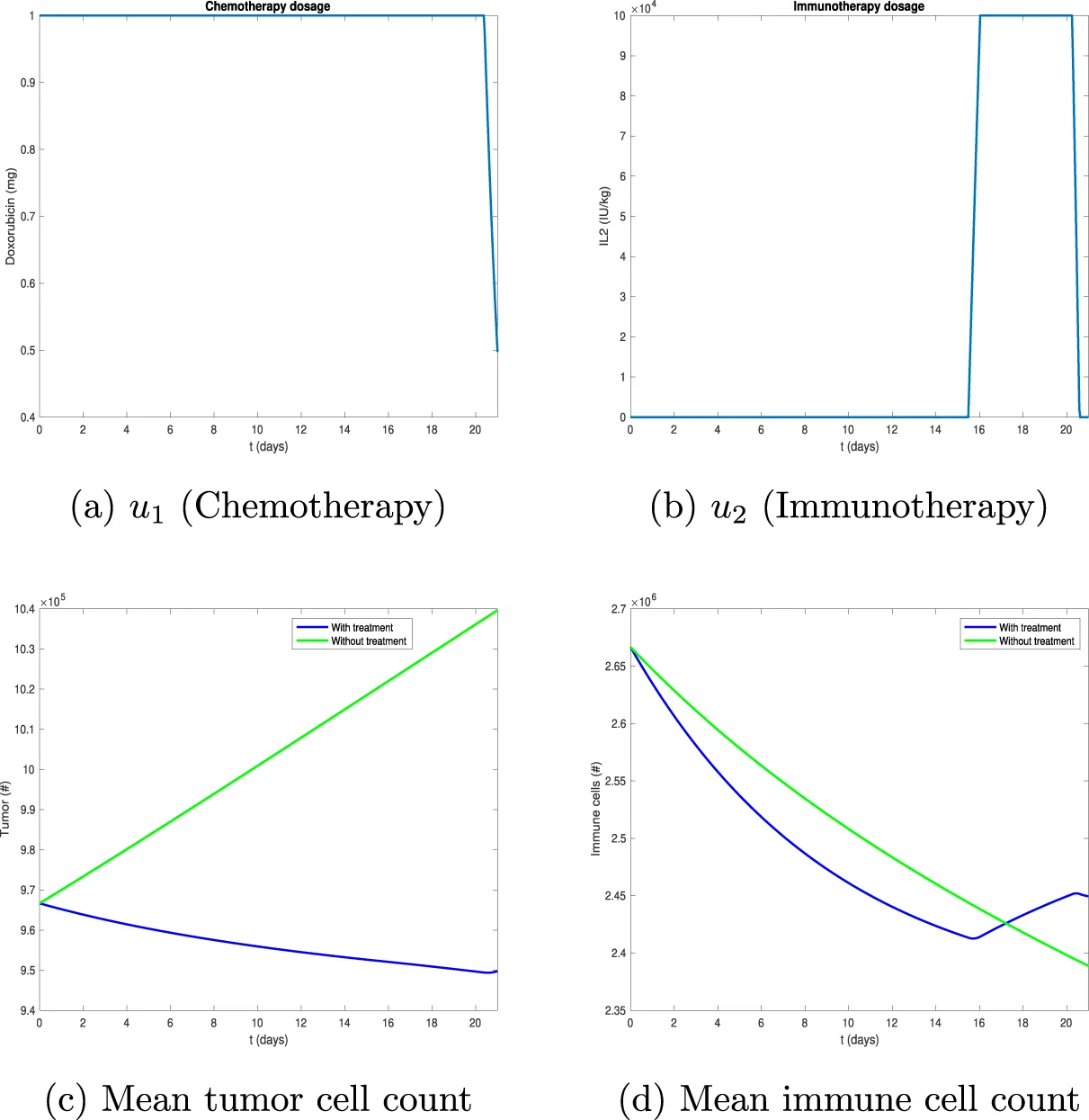
Test case 2: plots of the therapies. (a) $u_{1}$ (chemotherapy), (b) $u_{2}$ (immunotherapy), (c) mean tumour cell count and (d) mean immune cell count.

### 5.3. Limitations and future scope

The present study provides a first step towards a population-based Liouville framework for modelling heterogeneous cancer–immune dynamics and optimizing treatment in OC. Several limitations should, however, be acknowledged. First, the numerical experiments are based on synthetic population data rather than longitudinal clinical measurements from OC patients. Consequently, although the proposed framework demonstrates the feasibility of estimating population-level model parameters and designing treatment strategies in the presence of heterogeneous initial conditions, its predictive capability remains to be validated using patient data. Future studies will therefore focus on calibration and validation using longitudinal measurements of tumour burden, immune response and treatment outcomes from OC patient cohorts. Second, uncertainty in the present formulation is introduced through the initial cancer and immune cell populations, while the subsequent dynamics of each individual realization is governed by the deterministic pharmacokinetic model. Thus, stochastic fluctuations occurring during disease progression or treatment are not explicitly represented. An important extension will be to incorporate stochastic forcing into the underlying cancer–immune dynamics. This would lead naturally to a Fokker–Planck formulation and would allow both inter-patient variability in the initial state and dynamically evolving uncertainty to be modelled. Such an extension will require sufficiently informative longitudinal data to identify the associated drift and diffusion parameters. Finally, the OC pharmacokinetic model is intentionally reduced and represents chemotherapy and immunotherapy through their aggregate effects on the cancer and activated immune cell populations. Future work may incorporate additional biological mechanisms, including multiple immune cell populations, treatment resistance and patient-specific treatment response. These extensions, together with validation on clinical data, will enable the proposed population-based control framework to be investigated for the design of more individualized and clinically relevant treatment strategies.

## 6. Conclusion

In this work, a Liouville-based modelling and optimal control framework has been developed for describing heterogeneous cancer–immune dynamics in OC. Inter-patient variability is represented through a probability distribution of initial cancer and activated immune cell populations, whose evolution is governed by the Liouville equation while preserving the underlying deterministic cancer–immune dynamics. Within this framework, the parameters governing the principal cancer–immune interactions are first identified from population-level data using an adjoint-based projected NCG method, after which the time-dependent chemotherapy and immunotherapy dosages are optimized using a Pontryagin-based SQH method. Numerical experiments with synthetic population data demonstrate the ability of the framework to recover the underlying cancer–immune dynamics and determine treatment protocols that steer the population distribution towards a more favourable therapeutic state while accounting for treatment exposure. The principal contribution of this study is therefore the integration of mechanistic cancer–immune dynamics, population heterogeneity, parameter identification and treatment optimization within a unified framework, providing a mathematical foundation for population-based treatment design in OC and motivating future validation using longitudinal clinical data.

## Data Availability

Data and relevant code for this research work are stored in GitHub: http://github.com/roysouvik2/Liouville-esophageal-cancer-codes and have been archived within the Zenodo repository [52].
